# Potential of extracellular vesicle cargo as molecular signals in Schizophrenia: a scoping review

**DOI:** 10.1038/s41537-025-00566-5

**Published:** 2025-02-12

**Authors:** Shivaprakash Gangachannaiah, Smita Shenoy, Dinesh Upadhya, Elstin Anbu Raj Stanly, Nachiket Gudi, Pallavi Lakshmi Chandrashekar, Samir Kumar Praharaj

**Affiliations:** 1 Department of Pharmacology, Kasturba Medical College, Manipal, Manipal Academy of Higher Education, Manipal, 576104 Karnataka India; 2 Centre for Molecular Neuroscience, Kasturba Medical College, Manipal, Manipal Academy of Higher Education, Manipal, 576104 Karnataka India; 3 Centre for Evidence-informed Decision-making, Prasanna School of Public Health, Manipal, Manipal Academy of Higher Education, Manipal, 576104 Karnataka India; 4 Department of Physiology, Kasturba Medical College, Manipal, Manipal Academy of Higher Education, Manipal, 576104 Karnataka India; 5 Department of Psychiatry, Kasturba Medical College, Manipal, Manipal Academy of Higher Education, Manipal, 576104 Karnataka India

**Keywords:** Biomarkers, Schizophrenia

## Abstract

The diagnosis of schizophrenia (SCZ) primarily relies on clinical history and mental status assessments by trained professionals. There has been a search for biomarkers to facilitate laboratory diagnosis. Since extracellular vesicles (EVs) communicate with brain cells and can easily cross blood-brain barrier, there is increased interest among experts to explore them as potential molecular signals for disease detection. A scoping review was conducted to provide a comprehensive summary of the existing literature to identify the differentially expressed molecular signals in EVs isolated from SCZ patients. The methodological framework outline provided by Arksey and O’Malley was employed to conduct this scoping review. A systematic search was conducted using a search string across four databases, ultimately leading to selection of 24 relevant studies. Over 1122 differentially expressed biomolecules were identified in EVs extracted from biological fluids and tissues that can be primarily categorized as RNAs, proteins, and metabolites. Among them, 83 biomolecules were identified as validated differentially expressed molecular signals, which included metabolites, circRNAs, lncRNAs, miRNAs, and proteins. These biomolecules were found to affect cellular receptors and intracellular pathways, neurotransmitters, mitochondrial functions, immune-related functions, and metabolic pathways, which could serve as potential biomarkers for SCZ diagnosis.

## Introduction

Schizophrenia (SCZ) remains a major psychiatric disorder, contributing substantially to the global healthcare burden. Currently, 21 million people worldwide are diagnosed with SCZ^1^. It is estimated that 15.2 million people living with disability are attributed to SCZ, and the rate of all-cause mortality in SCZ over 15 years is estimated to be 2.08 times higher than in the general population^2^. Due to multiple causative factors and the limited knowledge of the pathogenesis of SCZ, the current treatment approach involves the use of antipsychotic drugs, with the primary objective being the alleviation of symptoms. Conventional management combines the administration of antipsychotic medications with psychosocial rehabilitation.

Early diagnosis and prompt treatment significantly improve outcomes and reduce relapse in SCZ^3^. However, diagnosis primarily relies on evaluating patient history and mental status by trained mental health professionals, which can be challenging due to symptom overlap and drugs mimicking clinical presentations. Nearly 25% of substance abuse cases initially present with psychosis. Misdiagnosis is common in children due to confusing and fragmented symptom presentation^4–6^. Disagreements among psychiatrists persist even after using DSM-5 criteria for the diagnosis of SCZ^7^. Furthermore, the current diagnostic methods fail to detect SCZ early, as clinical features develop much later than disease onset, leading to unsatisfactory symptom control, particularly the negative symptoms. Research has focused on identifying biomarkers for SCZ to guide personalized treatment. While brain tissue biopsies and cerebrospinal fluid (CSF) would best reveal underlying pathology, obtaining these from living patients poses ethical and safety concerns. Additionally, post-mortem sample evaluation may not detect the early changes in SCZ, considering the course of the disease.

Recent advancements in extracellular vesicles (EVs) have generated significant enthusiasm. EVs are lipid bilayered, cell derived vesicles, released by all types of cells in the body. They are categorized based on their size: apoptotic bodies (1000–5000 nm), microvesicles (MVs) (100–1000 nm), and exosomes (EXs) (50–150 nm). Microvesicles are membrane-bound entities that are released by outward budding from the plasma membrane of cells. Exosomes, on the other hand, are formed by inward budding of plasma membrane to form early endosome and late multivesicular bodies (MVBs) which fuse with the plasma membrane to release exosomes by the process of exocytosis^8^. Furthermore, apoptosis, the process of planned cell death, releases apoptotic bodies, another kind of cell-derived vesicle that may contain remains of the dying cell and cellular debris. EVs possess key attributes that are promising as biomarkers for mental health because they: 1) are engaged in cellular communication, 2) cross the blood-brain barrier (BBB), and 3) easily quantified from peripheral body fluids^9,10^. The ability to communicate between different cells, allows EVs to carry distinct disease-specific biomolecules from brain cells to peripheral blood. Characterizing peripheral blood EVs and studying their composition helps understand disease pathology.

EVs’ compositional diversity is noteworthy. A significant fraction of the proteins found in EVs are shared by different EV subtypes. These include cytoplasmic proteins, major histocompatibility complex (MHC) molecules, tetrameric proteins (CD9, CD63, CD81, and CD82), membrane transport and fusion proteins (GTPases, annexins, and flotillin), and heat shock proteins (Hsc70 and Hsp90). Their trafficking depends on the Endosomal Sorting Complex Required for Transport-3 (ESCRT-3) binding protein. Lipids such as phosphatidylcholine, phosphatidylethanolamine, sphingomyelin, phosphatidylserine, phosphatidylinositol, and monosialotetrahexosylganglioside (GM3), are abundantly present in the EV lipid bilayer^11^. EVs also contain nucleic acids like mRNA, mRNA fragments, long non-coding RNA (lncRNA), miRNA, ribosomal RNA (rRNA), distinct for different subtypes. Web-based resources like ExoCarta (http://www.exocarta.org/), Vesiclepedia (http://microvesicles.org/), or EVpedia (https://evpedia.info/evpedia2_xe/) provide comprehensive information on the proteins, lipids, and RNA content of EVs and EXs.

The techniques that are commonly used to isolate EVs include density gradient centrifugation, differential ultracentrifugation, size exclusion chromatography, ultrafiltration, immunomagnetic bead sorting, polyethylene glycol precipitation, and microfluidics. The characterization methods used for EVs include western blotting, flow cytometry, atomic force microscopy (AFM), transmission electron microscopy (TEM), resistive pulse sensing, nanoparticle tracking analysis, dynamic light scattering (DLS), and enzyme-linked immunosorbent assay (ELISA) (Supplementary Table S1)^12,13^.

However, rigorous characterization methods are needed to categorize EVs as MVs or EXs because of size overlap. Until live imaging technologies can capture real-time EV release by cells, the International Society for Extracellular Vesicles (ISEV) suggests adopting operational nomenclature^14^. Hence, in subsequent sections MVs and EXs are referred to as “EVs.”

Earlier studies from our laboratory have reported the benefits of characterizing and studying the composition of EVs for diagnosing and managing CNS and metabolic disorders such as epilepsy, Parkinson’s disease, and diabetic retinopathy^15–20^. The present review aims to screen the available literature to identify potential EV molecular signals for the early diagnosis and understand their role in disease pathophysiology.

## Methods

A scoping review methodology was considered appropriate to map emerging evidence on EV biomarkers and assess their diagnostic accuracy in SCZ. The review followed Arskey and O’Malley’s guidelines^21^, as recommended by JBI methods for scoping review. An a priori protocol was developed before the initiation of the study. The review is reported according to the Preferred Reporting Items for Systematic Reviews and Meta-Analyses extension for scoping reviews (PRISMA-ScR) guidelines (Supplementary Table 2)^22^.

### Identifying the research question

The research questions aimed to identify various EV molecular signals and explore their potential as objective laboratory evidence for diagnosing SCZ. This will help identify new biomarkers and better understanding of the affected physiological pathways, facilitating early diagnosis compared to currently used clinical criteria. The research questions were: What are the various potential EV molecular signals identified to date in SCZ? How can these marker signal help understand the disease pathogenesis?

### Identifying relevant studies

We did not use any specific frameworks such as PICO, CoCoPop, or PCC, as the research question does not fit into these classifications.

### Search strategy

The identified keywords “extracellular vesicles,” “schizophrenia,” “schizotypal personality disorder,” “schizophrenia, treatment-resistant,” “schizophrenia spectrum and other psychotic disorders,” “schizophrenia, paranoid,” “schizophrenia, disorganized,” “schizophrenia, childhood,” “schizophrenia, catatonic” were used in conjunction with Boolean operators such as AND / OR to form an appropriate search strategy for the individual databases. The search string used for PubMed (NCBI) is outlined in online supplement (Supplementary Table S3). The comprehensive search strategy was developed by EAR. The search was conducted across multiple databases, including 1) PubMed (NCBI), 2) Scopus (Elsevier), 3) CINAHL (EBSCO), and 4) EMBASE (Elsevier) by the search team (EAR, NG), and further validated by SP, PLC, DU. The search was restricted to articles published in the English language. The search included articles from inception of the respective database till Nov 2024, although all articles appeared from 2006 onwards.

### Study selection

All studies on the SCZ biomarkers were included except those in the grey literature, conference abstracts, preprints, and non-English articles (Table 1). Identified studies from database search were imported into Rayyan, a screening software. Two reviewers (S.P. and P.L.C.) independently conducted the screening in two sequential stages: Title-Abstract (Ti-Ab) and full-text screening. The articles were sorted into three categories in each stage: include, exclude, and maybe. Discussions with the third author helped to settle any differences between the two authors (S.K.P., S.S., and D.U.).

**Table 1:** Selection criteria.

Inclusion criteria	Exclusion criteria
English Literature	Non-English Literature
Peer-reviewed scientific literature	Non-peer reviewed literature and other animal studies, protocol papers, conference abstracts and pre-prints

### Charting the data

Two authors (S.P. and P.L.C.) used a predefined and validated data extraction sheet designed using Microsoft Excel 2019 (Microsoft Inc, Seattle, WA, USA). The identified domains for charting the data were study ID, study setting (country), study design, participant’s characteristics, drugs intake (Supplementary Table S4).

### Collating, analyzing, summarizing, and reporting the results

We analyzed the collected data and presented findings as percentages and frequencies. Google Spreadsheet and Microsoft Excel (Microsoft Inc., Seattle, WA, USA) were used to manage the data.

## Results

**Fig. 1:**
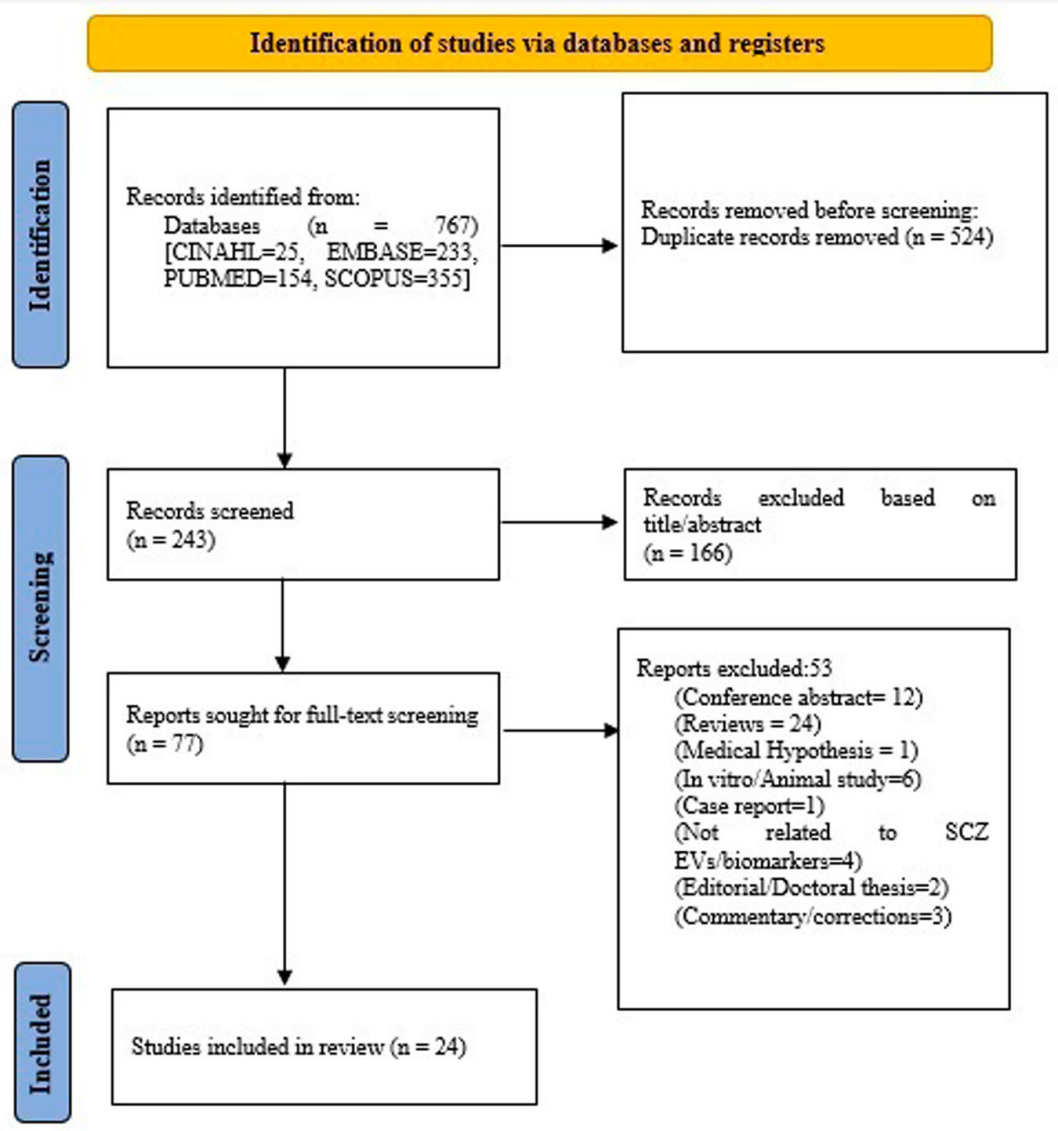
Flow chart of the study selection procedure. The figure outlines the PRISMA guideline’s systematic method for selecting studies, from initial identification to screening, eligibility evaluation, and inclusion, is shown in the flow chart. SCZ Schizophrenia, EV Extracellular vesicles, n Number.

A total of 243 studies were found for screening after excluding duplicates. After the title-abstract review, 77 full-text articles were assessed for inclusion and exclusion criteria. Following a full-text review and resolution of conflicts, 24 articles met inclusion criteria, as summarized in Fig. 1 and 53 studies were excluded (Supplementary Table S5). Most of the studies had a cross-sectional design except for seven, which had follow-up^23–29^. The duration of the illness ranged from 4 months to 26 years among the studies. Most of the studies were conducted in China (*n* = 8), followed by USA (*n* = 7), Japan (*n* = 2), Norway (*n* = 2), and one each in Greece, Germany, Spain, Switzerland, and Australia (Table 2 and Supplementary Table S4). Conflict of interest and funding source of studies are summarized in Supplementary Table S6.

**Table 2:** Characteristics of participants, extracellular vesicle, and analytical techniques used.

Author (Year)	Population character	EVs type, source, isolation and characterization	Analytical techniques
Banigan (2013)^30^	*N* = 30; *n* (cases)=8 (SCZ:47% & 53% BD); Mean age (Y): 69.87 ± 15.96; G: 100%M; Ethnicity: NR	Exosome EV source: Postmortem brain sample Ultracentrifugation method; Characterization: TEM, Immunogold labelling, western blotting	miRNA expression analysis by FlexMAP3D instrument by (Luminex Corporation, Austin, TX) and Luminex technology using oligonucleotide-Labeled beads to assay 312 miRNA; qPCR for specific miRNAs.
Amoah (2020)^31^	*N*: 80 *n* = 55 (SCZ 53%, BD 47%) Mean age(Y): 42.28 ± 1.59 G: 69% M Ethnicity: NR	Exosome EV source: Postmortem OFC Precipitation method using total exosome isolation buffer; Characterization: Exosome quantification with nanosight, electron microscopy characterization, and ELISA for CD63.	Total RNA isolated by miRNeasy RNA isolation kit. Mature miRNA reverse transcription with Taqman miRNA reverse transcription kit and qRT-PCR using Taqman miRNA assays.
Du (2019)^32^	**Training cohort** *N* = 46 *n* (SCZ) = 23; Mean age(Y): 27.4 ± 6.319 G:44% M **Testing cohort** *N* = 49, *n* (SCZ) = 26 Mean age(Y): 29.27 ± 5.311 G:50%M **Validation cohort** *N* = 200, *n* (SCZ) = 100 Mean age(Y): 29.77 ± 6.921 G:50%M Ethnicity: Chinese;	Exosome EV source: Serum Size exclusion chromatography(qEV); Characterization: Negative staining electron microscopy, nanoparticle tracking analysis and western blot for exosome markers	Exosomal RNA was isolated using the trizol. miRNA library construction and sequencing were performed at RiboBio (Guangzhou, China). Serum BDNF protein levels were measured by ELISA kit
Funahashi (2023)^34^	Microarray study: *N* = 18; *n* (SCZ) = 8 Mean age(Y): 41.9 ± 9.4 G: 44%M Replication set: *N* = 100 *n* (SCZ) = 50 Mean age(Y): 41.9 ± 9.4 G:48%M Ethnicity: Japanese	Exosome EV source: Plasma Ultracentrifugation method; Characterization by nanoparticle tracking analysis;western blot for protein markers	Total RNA was extracted using Trizol Reagent (Invitrogen, Carlsbad, CA, USA); Total RNA content was analysed using a bioanalyzer (Agilent Technologies); qPCR to examine miRNA expression by using the Mir-X miRNA First-Strand Synthesis Kit (Takara Bio Inc., Tokyo, Japan) and StepOne software (Applied Biosystems, Foster City, CA, USA); Bioinformatics analysis to confirm the target gene prediction of miRNA using the comprehensive miRNA database miRWalk (http://mirwalk.umm.uni-heidelberg.de/) (Dweep et al. Citation2011)
Tomita (2023)^24^	*N*: 30 *n* = 15 each in PPLE and RPLE group Mean age(Y): 13.91 ± 5.81 in PPLE 13.83 ± 5.27 in RPLE G: 60%M in each group Ethnicity: Asian	Exosome EV source: Urine Precipitation method: miRCURY Exosome Cell/ Urine/CSF Kit (QIAGEN, Hilden, Germany)	RNA extraction using a miRNeasy Micro kit (QIAGEN), Quality of extracted RNA using Bioanalyzer 2100 (Agilent, Santa Clara, CA, United States), miRNA-seq library was prepared using QIA-seq miRNA Library Kit (QIAGEN)
Tsoporis (2022)^23^	*N*: 24; *n* (SCZ) = 14 Mean age(Y): 24.5 ± 3.9; G: 55%M Ethnicity: NR	Exosome EV source: Plasma Spin-column based-SEC method; Characterization by NTA; Nanosight technology, electron microscopy and qRT-PCR for GT130, as a negative marker, and CD9, CD63, as positive marker	miRNA was isolated using Qiagen miRNeasy kit.
Khadimallah (2022)^35^	*N*: 272 *n*^a^ (EPP) = 138 (SCZ60.1% B D 10.1%, MDD 2.2%, SAD 8.7%, BPE 14.5%, NS 4.3%). Mean age(Y): 24.7 + 4.6 G: 74%M Ethnicity: Caucasian 66%; and others 34% (African, Asiatic, Metis)	Exosome Source: Plasma Precipitation-based method (ExoQuick Kit); immunostaining on a panel of exosomal proteins	miRNA extraction by miRNeasy Kit; reverse transcription using the miRCURY LNA Kit; qPCR amplification to quantifying miRNAs and measuring the COX6A2 protein by ELISA kit.
Barnett (2023)^33^	*N*:477 *n*(SCZ) = 221 Mean age(Y): 40 ± 10.7; G: 66.5%M Ethnicity: NR	Neuronal EVs EV source: Serum Immunoaffinity-based EV isolation method	RNA extraction: Trizol-based extraction method;SMARTer smRNA-seq kit is used to prepare small RNA libraries from total RNA; repared libraries were quantified using tools KAPA Biosystems (for quantification) and the Agilent small RNA chip (for size estimation).Sequencing is performed on the NovaSeq 6000 platform using the XP workflow.
Tan (2021)^37^	*N* = 22; *n* (SCZ) = 11 Mean age(Y): 34 ± 8.1 G:27%M; Ethnicity: Han	Exosome Source: Plasma Differential ultracentrifugation method; Charactarization by Nanosight LM10 and TEM	Total Exosomal RNA extraction by exoRNeasy Serum/Plasma Maxi Kit (Qiagen), RNA Quantification by Qubit 3.0, Library Construction Epibiotek (Guangzhou, China); circRNA sequencing by Illumina HiSeq and identification using a computational pipeline ACFS2,differential expression analysis by DEGseq Software.
Xinzhe Du (2023)^36^	*N*:20 *n*(SCZ) = 10 Mean age(Y): 34.2 ± 12.01 G: 50%M Ethnicity: Hans Chinese	Exosomes Source: Plasma Membrane-based affinity isolation	RNA isolation-NEBNext® rRNA depletion kit; RNA library preparation using the truSeq stranded kit; RNA sequencing by Next-generation sequencing (NGS) using the Illumina NovaSeq 6000 with paired-end sequencing; prediction tools (MiRmap, microT, miRanda, PicTar, TargetScan) were used to identify which miRNAs regulate mRNAs. The resulting relationships were then visualized as a network using cytoscape.
Guo (2022)^27^	*N*: 152 *n* (SCZ) = 56 G: 52%M Mean age(Y): 31.38 ± 6.378 Ethnicity: NR	Exosome Source: Serum Isolation and characterization-NR	**NR**
Ranganathan (2022)^38^	*N*: 36; *n* (SCZ) = 24 Median age(Y): 26 (4.25) median(IQR) G: 83%M Ethnicity: Caucasian 7 African American 11 Other 6 Substance abuse among cases: Nicotine (54%); cannabies (29%) in last 30 days	Exosome Source: Plasma Precipitation method by standard exosome isolation kit. Characterization by Nanosight	Western blot for protein markers of exosome and proteins of interest GFAP and α-II-Spectrin.
Lee (2021)^39^	*N*: 120; *n* (SCZ) = 60 Mean age(Y): 48.3 ± 10.3 G: 45%M Ethnicity: Caucasian (37%), Hispanic (33%), & others (30%) Substance abuse among cases: Alcohol (48%); Smokers+	Exosome Source: Plasma Precipitation method by ExoQuick. Characterization by nanoparticle tracking analysis, TEM and western blot for exosome marker	Quantification of NDE and ADE Protein Cargo by ELISAs using the Pierce BCA Protein Assay kit (Thermo Fisher Scientific; Catalog # 23225)
Goetzl (2022)^40^	*N*: 20; *n*^a^ =10 Mean age(Y): 21.5 ± 3.28 G: 70%M Ethnicity: NR	Exosome Source: Plasma Precipitation-based (ExoQuick-System Biosciences, Mountain View, CA); Characterization by nanoparticle tracking analysis	ELISA kits for protein quantification.
Goetzl (2021)^41^	*N*: 20; *n*^a^ =10 Mean age(Y): 21.5 ± 3.28 G: 70%M Ethnicity: NR	Exosome Source: Plasma Precipitation-based ExoQuick-System. Characterization by nanoparticle tracking analysis	ELISA kits for protein quantification.
Goetzl (2020)^42^	*N*: 20; *n*^a^=10 Mean age(Y): 21.5 ± 3.28 G: 70%M Ethnicity: NR	Exosome Source: Plasma Precipitation-based (ExoQuick-System Biosciences, Mountain View, CA); Characterization by nanoparticle tracking analysis	ELISA kits for protein quantification.
Kapogiannis (2019)^43^	*N* = 48; *n* (Drug Naïve First Episode Schizophrenia)=24 Mean age(Y): 32.75 ± 11.76; G:M54% Ethnicity: NR	Neuronal EVs EV source: Plasma Precipitation-based (ExoQuick-System Biosciences); Characterization by Nanosight NS500 (Malvern).	Protein quantified by electrochemiluminescence (MesoScale diagnostics).
Tunset (2020)^26^	*N*: 50; *n*^a^ (First Episode Psychosis or acute psychosis) =25 (SCZ 48%, substance-induced 16%, acute psychosis 12%, unspecified psychosis 12%,other psychotic disorders 12%) Mean age(Y): 33.1 ± 11.0 G: 76%M Ethnicity: NR	EVs Source: Plasma Differential centrifugation; Characterization by NTA; protein concentration was determined by Qubit Quant-IT Protein Assay Kit.	Protein composition of EVs was determined by LC-MS/MS analysis.
Ting Xue (2024)^28^	*N*:343;*n* = 134(18% BD; 13% MDD) Mean age(Y): SCZ(Mean ± SD) Set 1- 38.83 ± 12.29; Set 2-38.00 ± 13.2; FEDN 30.18 ± 10.78 G: 40%M(Set-1); 50%M(Set-2); 53%M(Set-3) Ethnicity: Han Chinese	EVs Source: Plasma Ultracentrifugation; western blot for EV markers and complement activation; nanoparticle tracking analysis and TEM	Plasma-derived complement DEPs were quantified by ELISA; EVs-derived complement DEPs were detected and quantified by Mesoscale Discovery electrochemiluminescence assays (MSD, Maryland, US). Proteomics analysis of EVs by Synapt G2-Si quadrupole time-of-flight mass spectrometer equipped with ion mobility option (Waters Corporation). XG Boost algorithm to construct Personalized Discrimination Score(PDS)
Cristina Lorca (2024)^45^	*N*: 30; *n* = 15 Mean age(Y): 52.08 ± 7.02 G: 80%M Ethnicity: NR	EVs EV source: Postmortem brain (Prefrontal cortex, Hippocampus, Caudate) Brain enrichment of EVs by Protein Organic Solvent Precipitation (PROSPR); Characterization of Brain EVs using nanoparticle tracking analysis and TEM	Lebel-free proteomics by Liquid Chromatography-Mass Spectrometry (LC-MS/MS) using the EVOSEP liquid chromatographer at 300 nL/min with an 88-minute gradient and analyzed by using four-dimensional (4D) parallel accumulation–serial fragmentation (PASEF) data acquisition.
Tunset (2023)^25^	*N*: 50; *n*^a^ (First Episode Psychosis or acute psychosis)=25 (SCZ 48%, substance-induced 16%, acute psychosis 12%, unspecified psychosis 12%,other psychotic disorders 12%) Mean age(Y): 33.1 ± 11.0 G: 76%M Ethnicity: NR	EVs Source: Plasma Differential centrifugation; Characterization by nanoparticle tracking analysis	Proteomic analysis by metaproteomic data analysis; Protein composition of EVs was determined by LC-MS/MS analysis; LPS content in isolated EV was determined by the PyroGene recombinant factor C endotoxin detection end-point assay (Lonza, Belgium)
Zhang S (2024)^29^	*N*: 50; *n* (RES = 10 and NRES = 10). Mean age(Y): 20.50 ± 3.92 RES; 19.50 ± 5.42 NRES G: 40%M for RES;60% for NRES; Ethnicity: Han Chinese	Exosome Source: Plasma SEC with Ultrafiltration; Characterization by protein markers (TSG101 + /HSP70 + /CD9 + /Calnexin-) and transmission electron Microscope (H-7650, Hitachi, Japan)	Proximity barcoding assay (PBA) was performed in Echobiotech (Beijing, China). The surface proteins on individual EVs were profiled using the proximity barcoding assay ExoSeek® panel260 (Secretech, Shenzhen, China)
Du (2021)^46^	**Training set** *N* = 144, *n* (SCZ) = 78; Mean age(Y) = 31.7 ± 7.73; G:86%M; **Testing set 1** *N* = 169, *n*(SCZ) = 107; Mean age(Y) = 31.45 ± 8.85; G:80%M; **Testing set 2** *N* = 246, *n*(SCZ) = 104; Mean age(Y) = 26.69 ± 8.2; G:51.92%M; **Testing set 3** *N* = 158, *n*(SCZ) = 96; Mean age(Y) = 29.98 ± 5.76; G:52.08%M; Ethnicity: Han Chinese & Uyghur Chinese	Exosome Source: Serum SEC; Characterization by negative-staining electron microscopy, nanoparticle tracking analysis, and western blotting	Targeted metabolomics using UPLC (Shim-pack UFLC SHIMADZU CBM30A system) and tandem mass spectrometer (MS/MS) (4500 QTRAP; Applied Biosystems) equipment.
Xu CX (2024)^47^	*N*: 40; *n* (First Episode schizophrenia and drug free)=22 Mean age(Y): 26.3 ± 6.30 G: 50%M Ethnicity: NR	Exosome Source: Serum SEC; Characterization by negative-staining electron microscopy, nanoparticle tracking analysis, and western blotting	Targeted lipidomics by UPLC and tandem mass spectrometry (MS/MS) (4500 QTRAP; Applied Biosystems) equipment. Qualitative analysis Metware. Qantified lipids via multiple reaction monitoring via triple quadrupole mass spectrometry.

*NR* not reported; *N* Total sample, *n* cases, *Y* years, *EV* Extracellular vesicles, *SCZ* Schizophrenia, *MDD* Major Depressive Disorder, *BD* Bipolar Disorder, *G* Gender, *M* Male, *PM brain sample* Post mortem brain sample, *Postmortem OFC* Postmortem brain sample orbitofrontal cortex, *PPLE* Persistent psychotic like episode group, *RPLE* Recurrent psychotic like episode group, *EPP* Early psychosis patients, *BDNF* Brain-derived neurotrophic factor, *GFAP* glial fibrillary acid protein, *SAD* Schizo Affective Disorder, *BPE* Brief psychotic episode, *NS* Nonspecified psychosis, *ADE* Astrocyte derived exosomes, *NDE* Neuron derived exosomes, *RES* Antipsychotic responders, *NRES* nonresponders, *SEC* Size Exclusion Chromatography, *SCZ* Schizophrenia.

^a^First episode psychosis.

From the pooled studies over 1122 potential signal markers were identified in EVs extracted from biological fluids, categorized mainly as **genetic signals, protein signals**, and **other metabolomic signals**.

### Genetic factors

**Fig. 2:**
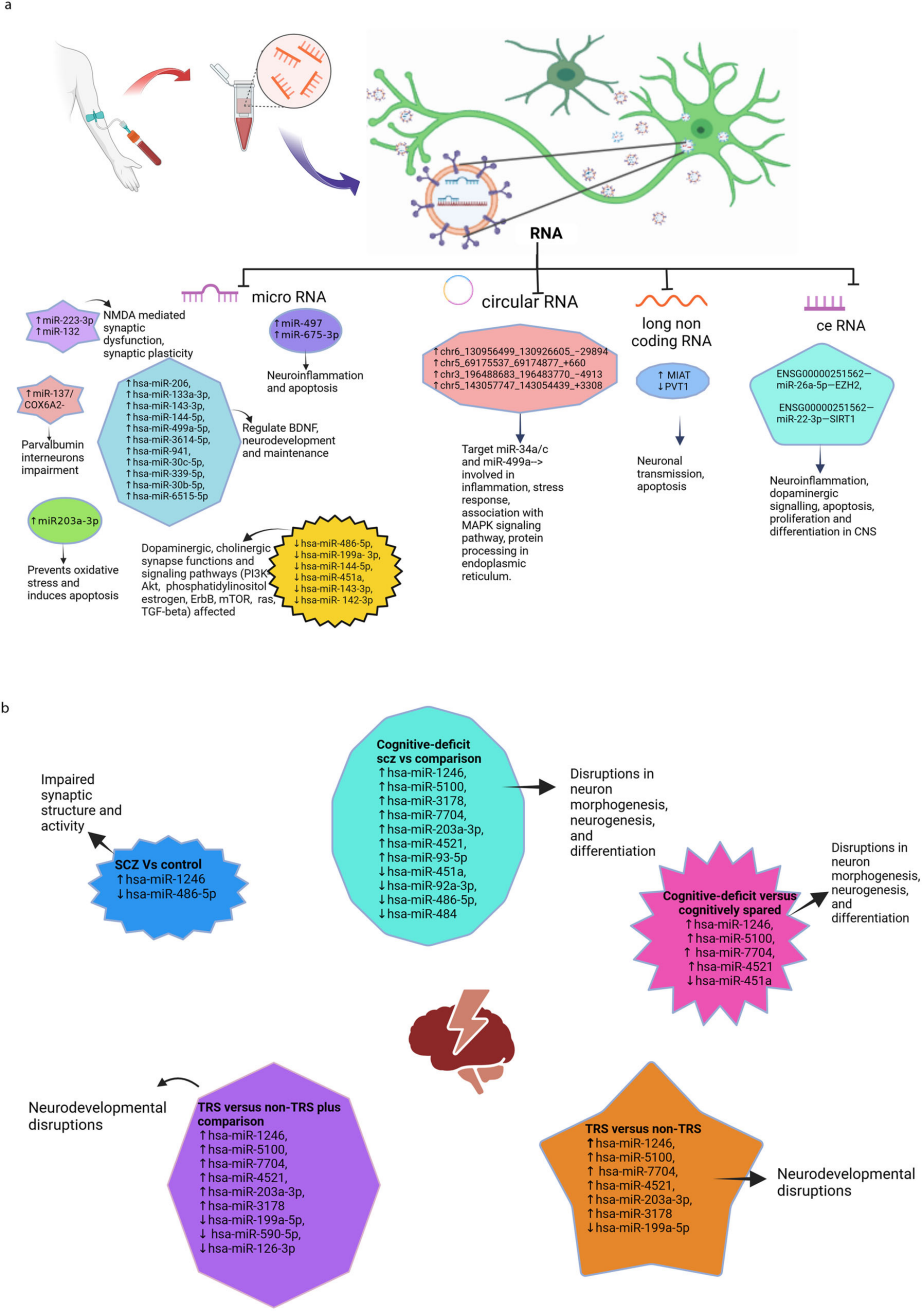
Changes in RNA types associated with schizophrenia. **a** Differentially expressed RNAs in schizophrenia and their associated functions. **b** Differentially expressed miRNAs in schizophrenia cases, subgroups and their associated functions. NMDA N-methyl-D-aspartate, SCZ schizophrenia, BDNF brain-derived neurotrophic factor, TRS treatment-resistant cases of schizophrenia, Non-TRS non-treatment-resistant cases of schizophrenia, PI3K-Akt phosphatidylinositol 3-Kinase - Protein Kinase B (Akt) signalling pathway, mTOR mammalian target of rapamycin, ceRNA network RNA (lncRNA-miRNA-mRNA), MAPK mitogen-activated protein kinase, MIAT myocardial infarction–associated transcript, PVT1 plasmacytoma variant translocation 1, TGF-β transforming growth factor-beta.

Eleven studies identified differentially expressed RNAs, including microRNAs, circular RNAs (circ-RNA), long non-coding RNAs (lncRNAs), and mRNAs as summarized in Fig. 2a, b.

### MicroRNAs

The studies found 59 differentially expressed miRNAs. A study showed differential expression of two separate miRNAs aiding in diagnosing SCZ^30^. In prefrontal cortex brain samples, miR-497 was specifically upregulated in SCZ patients (*P* < 0.05) but not in bipolar disorder (BD). On the other hand, miR-29c was increased explicitly in BD (*P* < 0.05) but not in the SCZ group. Similarly, in another study^31^, in orbitofrontal cortex postmortem brain sample of 29 SCZ and 26 BD cases, miR-223-3p was raised and miR-132 decreased in SCZ, but not in BD. Further, the study showed a negative correlation of miR-223 on its target mRNAs glutamate ionotropic receptor AMPA-type subunit 2 (*GRIA2*) and glutamate ionotropic receptor NMDA-type subunit 2B (*GRIN2b*), and a positive correlation with inflammation associated *SERPINA3* mRNA. Few mi-RNAs, such as miR-193b-3p and miR-28a-3p, were increased in both SCZ and BD compared to controls.

MicroR-206, which regulates expression of brain-derived neurotrophic factor (BDNF) was increased in SCZ and validated by qRT-PCR^32^. Further, ten other microRNAs were found to be differentially expressed in SCZ and were found to target genes involved in inflammation and apoptosis. A study reported 2 miRNAs differentially expressed in SCZ compared to nonpsychiatric group: hsa-miR-1246 (upregulated) and hsa-miR-486-5p (downregulated). Furthermore, 6 miRNAs (hsa-miR-1246, hsa-miR-5100, hsa-miR-3178, hsa-miR-7704, hsa-miR-203a-3p, hsa-miR-4521) were upregulated and 5 miRNAs, (hsa-miR-451a,hsa-miR-92a-3p, hsa-miR-486-5p, hsa-miR-484, hsa-miR-93-5p) downregulated in cognitive deficit SCZ compared to non-psychiatric comparison groups (Fig. 2b)^33^.

Few candidate miRNAs were found to differentiate the subset group among SCZ patients. A study detected elevated expression of miR-675-3p in treatment-resistant cases (TRS) of SCZ compared to non-treatment-resistant cases (non-TRS)^34^. MicroR-675-3p was found to target mRNA regulating apoptosis and inflammation. Similarly, a set of six miRNAs was downregulated in EVs extracted from urine in subjects with persistent psychotic-like experiences, compared to the remitted group^24^. The pathways involved were identified by KEGG enrichment pathway analysis and were found to affect dopaminergic synapses. The average AUC for the combined six miRNAs was 0.847 (95% CI 0.690–0.994). In another study, hsa-miR-1246, hsa-miR-451a, hsa-miR-5100, hsa-miR-7704, hsa-miR-4521 were found to differentiate cognition deficit subset from cognition spared SCZ cases. Also, the same miRNAs, together with hsa-miR-590-5p and hsa-miR-126-3p, were shown to be differentially expressed when TRS was compared to non-psychiatric comparator^33^. A study detected increased miR- 203A-3P with decreased DJ-1 mRNA in SCZ cases compared to matched controls^23^. The translated DJ-1 protein was found to act as a scavenger for oxidative stress and was associated with apoptosis by bioinformatic analysis. However, following treatment with olanzapine, the levels miR-203 were reduced, similar to control levels.

Khadimallah et al.^35^ detected upregulated miR-137 levels coupled to reduced Cox 6 A levels in early psychosis patients compared to healthy controls. Increased expression of miR-137 was found to induce mitochondrial damage. These changes in combined miR-137/COX6A2 plasma EV levels (increased miR-137 and reduced COX6A2) were found to represent a proxy marker of parvalbumin interneurons impairment, which are critically involved in SCZ psychopathology and cognition.

In a large-scale comprehensive analysis of plasma exosomes in first episode SCZ Du X et al., identified 690 upregulated and 1947 downregulated mRNAs, 14 upregulated and 8 downregulated miRNAs, 385 upregulated and 361 down regulated lnc RNAs compared to healthy controls. A ceRNA network was built on highly intersected lncRNA-miRNA-mRNA which included one lncRNA, 2miRNAs (miR-26a-5p and miR-22-3p) and 2 mRNA (EZH2 and SIRT1) and concluded that network have the potential as diagnostic molecular signal in early schizophrenia. The AUC of network ENSG00000251562—miR-26a-5p—EZH2 (AUC = 0.85), ENSG00000251562—miR-22-3p—SIRT1 (AUC = 0.87), were found to be higher than lncRNA, miRNA and mRNA alone (Fig. 2a)^36^.

### Long non-coding RNA

Long non-coding RNA (lncRNA) myocardial infarction–associated transcript (MIAT) was increased, and plasmacytoma variant translocation 1 (PVT1) was reduced in SCZ cases^27^. The discriminatory ability of the differentially expressed molecular signals was confirmed by performing ROC analysis.

### Circular RNA

A set of four circular RNAs (circRNAs) were detected to be expressed more in SCZ cases compared to healthy controls^37^. They were found to target miR‐34a/c and miR‐499a, which are involved in the pathogenesis of SCZ. The differentially expressed circRNAs were validated by qRT-PCR in a fresh set of six SCZ patients compared to controls.

### Proteins

**Fig. 3:**
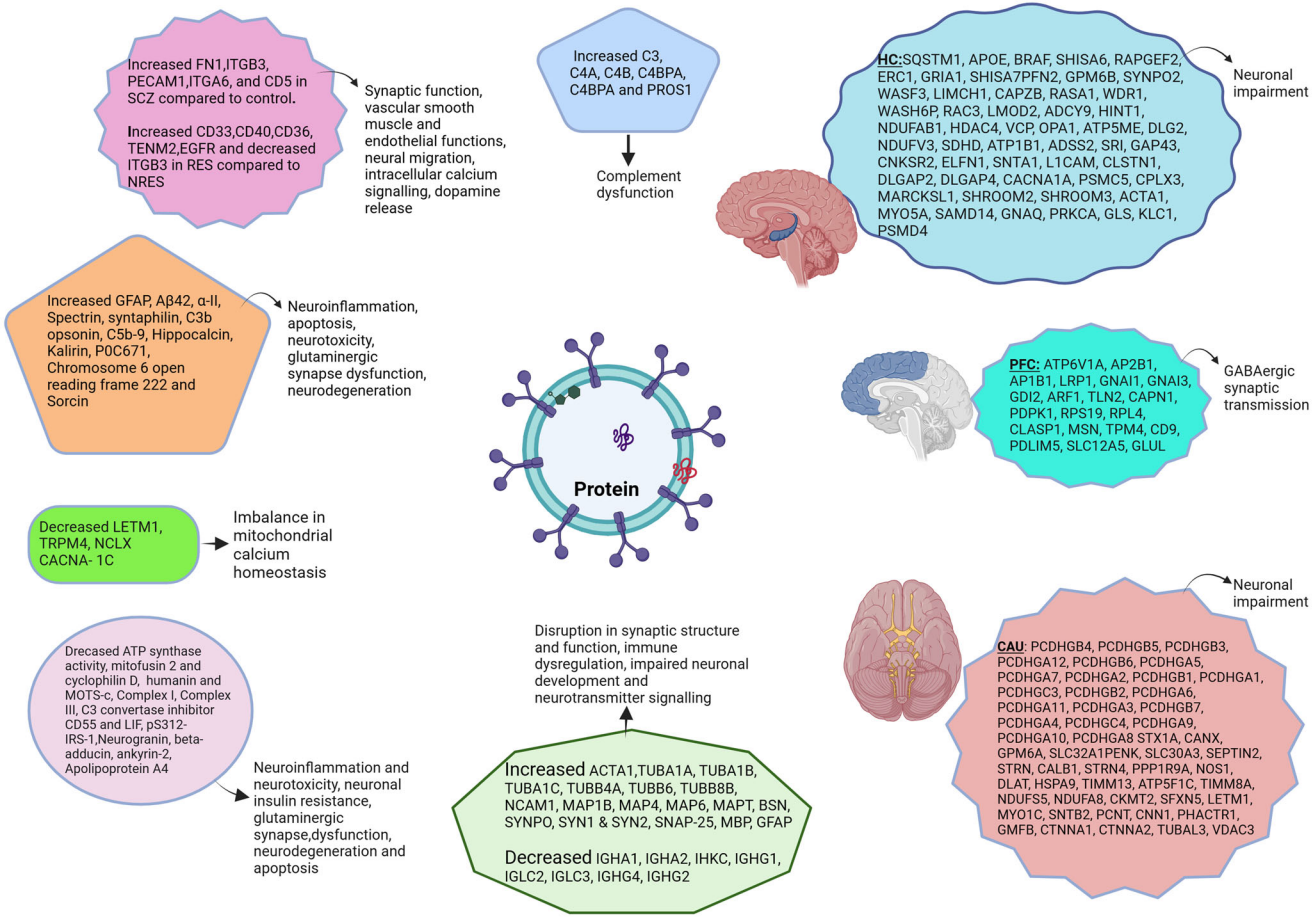
Changes in proteins related to schizophrenia. Differentially expressed proteins in schizophrenia and their associated functions. SCZ schizophrenia, RES antipsychotic responders, NRES antipsychotic nonresponders, GFAP glial fibrillary acidic protein, Aβ42 amyloid-beta 1-42, LETM1 leucine zipper EF-hand containing transmembrane 1 protein, TRPM4 transient receptor potential cation channel subfamily M member 4, NCLX mitochondrial Na+/Ca++ exchanger, CACNA-1C mitochondrial voltage-dependent L-type calcium channel subunit α- 1C, MOTS-c mitochondrial open-reading frames of the 12S rRNA-c, LIF leukemia inhibitory factor, pS312-IRS-1 phosphorylation at serine 312 of insulin receptor substrate 1, GABA Gamma-aminobutyric acid, HC hippocampus, PFC prefrontal cortex, CAU caudate.

The screened studies identified over 241 differentially expressed proteomic signal molecules. Proteins like Glial Fibrillary Acidic Protein (GFAP), associated with astrocytic activation and neuroinflammation, were up-regulated, and alpha-2-spectrin levels associated with neuronal loss, were decreased in SCZ^38^. Amyloid-beta 1-42 (Aβ42), was increased in SCZ cases compared to the control group^39^. Levels of mitochondrial proteins including leucine zipper EF-hand containing transmembrane 1 protein (LETM1), transient receptor potential cation channel subfamily M, member 4 (TRP M4), mitochondrial Na + /Ca + + exchanger (NCLX) were lower, whereas mitochondrial voltage-dependent L-type calcium channel subunit α- 1 C (CACNA-1C) were higher in those with first-episode psychosis^40^. Levels of mitofusin 2 (MFM 2), cyclophilin D (CYPD), and ATP synthetase activity were decreased, while the syntaphilin (SNPH) peptide involved in the maintenance of ATP levels was found to be increased. Humanin and mitochondrial open-reading frames of the 12S rRNA-c (MOTS-c) levels were significantly lower in first-episode cases^41^. Mitochondrial complex proteins like Complex I and Complex III were decreased in EVs of first-episode psychotic patients compared to controls, whereas GFAP was higher. The study also detected alteration in proteins involved in the immune system in SCZ. Complement proteins such as C3b and C5b-9 were higher, whereas C3 convertase inhibitor CD55 levels were lower. When compared to controls, first-episode psychosis (FEP) patients had significantly decreased levels of the neuroprotective protein leukemia inhibitory factor (LIF)^42^. Similarly, Zue T et al., reported increase in complement proteins C3, C4A, C4B, C4BPA, C4BPB and PROS1 in SCZ compared to healthy control (Fig. 3)^28^.

A study reported 52 differentially expressed proteins (DEPs) in SCZ compared to HC and 18 DEPs in antipsychotic responders (RES) compared to nonresponders (NRES). The study identified, increased 5 SCZ related proteins FN1,ITGB3, PECAM1, ITGA6, and CD5. Furthermore, the study found 6 altered SCZ proteins incuding increased CD33, CD40, CD36, TENM2, EGFR, and decreased ITGB3 DEPs in RES compared to NRES^29^.

In another study, abnormalities in neuronal insulin signaling were found in drug- naïve first-episode SCZ cases, leading to decreased signal transduction protein biomarker pS312-IRS-1^43,44^. Five brain proteins involved in the glutamatergic synapses were found to be differentially expressed in psychotic patients compared to healthy controls. Levels of hippocalcin and kalirin increased, while neurogranin, beta-adducin, and ankyrin-2 were reduced^26^. A study reported reduced EV levels of intestinal protein Apolipoprotein A4 (APOA4), and rised P0C671 Chromosome 6 open reading frame 222 (C6orf222), sorcin proteins in psychotic patients^25^.

Lorca et al. ^45^ reported differentially expressed unique proteins. There were 19 downregulated structural proteins: Actin (ACTA1), tubulins (TUBA1A, TUBA1B, TUBA1C, TUBB4A, TUBB6, and TUBB8B), cell adhesion (NCAM1), and microtubule-linked proteins (MAP1B, MAP4, MAP6, and MAPT), bassoon (BSN), synaptopodin (SYNPO), synapsins (SYN1 and SYN2), SNAP 25, myelin protein (MBP), astroglial marker (GFAP) and 8 upregulated immunoglobulins: IGHA1, IGHA2, IHKC, IGHG1, IGLC2,IGLC3, IGHG4, IGHG2. These changes were primarily observed only in the prefrontal cortex (PFC) region. Additionally, the study found 127 unique proteins in SCZ cases distributed in three brain regions PFC, hippocampus (HC) and caudate (Fig. 3)^45^.

### Metabolites

**Fig. 4:**
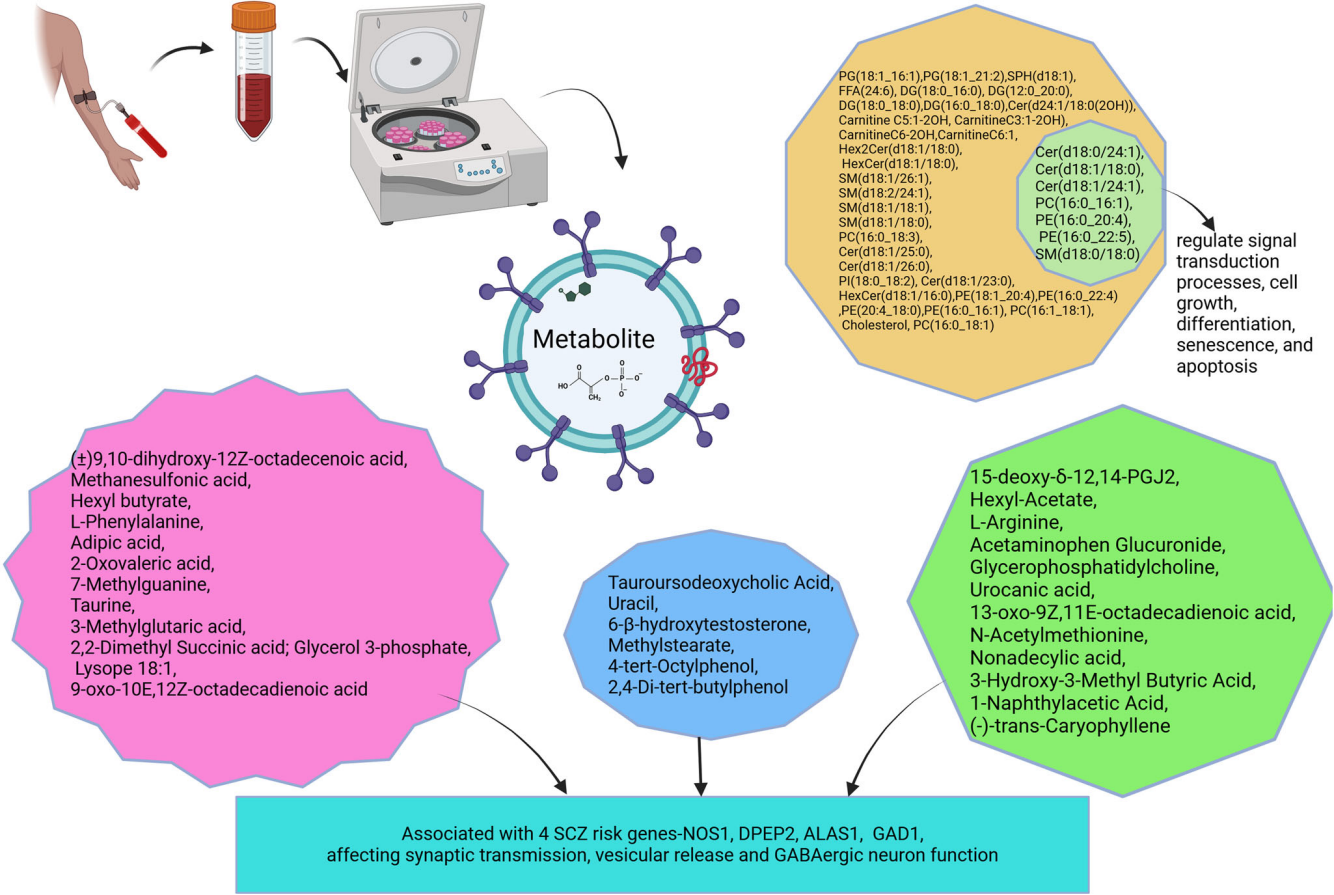
Changes in metabolites related to schizophrenia. Differentially estimated metabolites in schizophrenia and their associated functions SCZ schizophrenia, GABA gamma-aminobutyric acid, PCs phosphatidylcholines, PEs phosphatidylethanolamines, PS phosphatidylserine, PI phosphatidylinositol, SMs sphingomyelins, Cer ceramides, HexCer hexaceramides, PG phosphatidylglycerol, FFA free fatty acids, SPH sphingosine, DG diacylglyceride.

Compared to healthy controls, there was a significant difference in the estimated quantities of 25 metabolites (10 elevated and 15 decreased) in serum isolated EVs of SCZ patients^46^. KEGG pathway enrichment analysis found that these metabolites were associated with pathways related to glycerophospholipid metabolism and the biosynthesis of phenylalanine, tyrosine, and tryptophan. Taurine and l-arginine were functionally connected to four SCZ risk genes. Validity was confirmed at the analysis level by multi-correlation coefficient analysis to assess the relationship between the 25 metabolites and potential confounders, such as age and disease severity. Further, six metabolites distinguished between first-episode, drug-free, and chronically treated patients with SCZ patients (Fig. 4).

A study reported 39 differential metabolites in first episode drug free SCZ cases compared to healthy control. The study found seven among them have strong diagnostic potential in early SCZ^47^.

### Validation of candidate molecular signals

Validation is a process used to establish its performance that is acceptable for its intended purpose. The present study identified 1122 potential candidates in SCZ, of which 83 were found to be validated (Table 3). Four studies in particular reported 50 signal markers that had been validated both internally and externally^28,32,37,46^.

**Table 3:** Validated potential molecular signals of schizophrenia.

Biomolecules		Validation	Potential use
MicroRNAs	miR-497, miR-223-3p, miR-132, miR-675-3p^a^, miR-486-5p, miR-199a-3p, miR-144-5p, miR-451a, miR-143-3p, miR- 142-3p, miR-206, miR-133a-3p, miR-143-3p, miR-144-5p, miR-499a-5p, miR-3614-5p, miR-941, miR-30c-5p, miR-339-5p, miR-30b-5p, miR-6515-5p.	qPCR qRT-PCR qPCR Average AUC was 0.847 (95% CI 0.690–0.994) for combined 6 miR in ROC qRTPCR in fresh set of SCZ gp. A cluster of 11 micro-RNA showed AUC of 0.753 (95% CI, 0.61 to 0.90) in ROC.	DI DI and predictive for therapy response DI and predictive for therapy response. Disease predictive marker. DI
Combined MicroRNA with protein	miR-137 with COX6A2	AUC of Psy-D group (0.96) was higher than that of both Psy-ND (0.63) and Control subjects (0.74). Combined detection of miR-137 and COX6A2 protein levels detected Psy-D patients, with the highest sensitivity and specificity.	Diagnostic for early diagnosis and prognostic marker for disease severity
Circular RNAs	chr6_130956499_130926605_ − 29894, chr5_69175537_69174877_ + 660, chr3_196488683_196483770_ − 4913, chr5_143057747_143054439_ + 3308	qRTPCR in 6 fresh set of SCZ patients	DI
lncRNA	MIAT, PVT1	AUC for MIAT, PVT1 and combined was 0.897, 0.710, and 0.920, respectively	DI
ceRNA	ENSG00000251562—miR-26a-5p—EZH2 ENSG00000251562—miR-22-3p—SIRT1	AUC = 0.85 and AUC = 0.87 in ROC	Early diagnostic marker and prognostic for disease severity
Proteins	C3, C4, C4BPA, PROS1	Validated using MSD technology in independent set of 26 patients and 26 healthy controls. Four indexes performed better in distinguishing between chronic and first-episode drug-naïve (FEDN) patients, with a 0.881 AUC, 82.2% accuracy, 85.4% sensitivity, and 78.7% specificity.	DI and predictive for therapy response
	Altered proteins in SCZ Vs healthy control: FN1, ITGB3, PECAM1, ITGA6, CD5 Altered proteins in RES vs NRES: CD33, CD40, CD36, TENM2, EGFR and decreased ITGB3	ROC curves in distinguishing SCZ Vs healthy control: FN1(AUC = 0.805); ITGB3(AUC = 0.75); PECAM1(AUC = 0.8); ITGA6(AUC = 0.73) CD5(AUC = 0.8) ROC curves in distinguishing RES and NRES: ITGB3(AUC = 0.77), CD33(AUC = 0.87), CD40(AUC = 0.79), CD36(AUC = 0.81), TENM2(AUC = 0.86), EGFR(AUC = 0.85)	DI DI and predictive for therapy response
Metabolites	( ± )9,10-dihydroxy-12Z-octadecenoic acid; Methanesulfonic acid; Hexyl butyrate; L-Phenylalanine; Adipic acid; 2-Oxovaleric acid; 7-Methylguanine; Taurine; 3-Methylglutaric acid; 2,2-Dimethyl Succinic acid; Glycerol 3-phosphate; Lysope 18:1; 9-oxo-10E,12Z-octadecadienoic acid; 15-deoxy-δ-12,14-PGJ2; Hexyl-Acetate; L-Arginine; Acetaminophen Glucuronide; Glycerophosphatidylcholine; Urocanic acid; 13-oxo-9Z,11E-octadecadienoic acid; N-Acetylmethionine; Nonadecylic acid; 3-Hydroxy-3-Methyl Butyric Acid; 1-Naphthylacetic Acid; (-)-trans-Caryophyllene; Tauroursodeoxycholic Acid; Uracil; 6-β-hydroxytestosterone; Methylstearate; 4-tert-Octylphenol; 2,4-Di-tert-butylphenol.	Validity was confirmed by additional 3 test sets and at analysis level by multi-correlation coefficient analysis. An ROC curve in training set yielded AUC of 95.7% (95% CI = 92.6%–98.9%). Metabolites showed good differentiation between patients and controls in the 3 independent groups with accuracies: 91.0% (95% CI: 85.7%–96.3%), 82.7% (95% CI: 77.6%–87.9%), and 99.0% (95% CI: 97.7%–100%).	DI
	**Lipids** Cer(d18:0/24:1), Cer(d18:1/18:0), Cer(d18:1/24:1), PC(16:0_16:1), PE(16:0_20:4), PE(16:0_22:5), and SM(d18:0/18:0). Combined seven lipids	AUCs in ROC curves were 0.875 (95%CI = 0.752–0.973), 0.853 (95% CI = 0.699–0.958), 0.807(95% CI = 0.661–0.941), 0.858 (95% CI = 0.7–0.968), 0.807(95% CI = 0.66–0.933), 0.82 (95% CI = 0.655–0.938), and 0.842 (95% CI = 0.688–0.95). Overall analysis of these seven lipids 0.94 (95% CI = 0.82–1)	Diagnostic and prognostic potential to disease severity.

*DI* Diagnostic indicator, *PVT1* plasmacytoma variant translocation 1, *MIAT* Myocardial infarction–associated transcript, *Psy-D* group with mitochondrial dysfunction, *Psy-ND* group with no/low mitochondrial impairment, *AUC* Area under the curve, *ROC* Receiver operating characteristic curve, *MSD* Mesoscale Discovery electrochemiluminescence assays.

^a^The study findings need to be confirmed further in an appropriate comparator population^86^.

## Discussion

This scoping review found over 1031 potential signal molecules from the included studies. In most studies, EVs were isolated from plasma (*n* = 14); in four studies, serum was the source; brain tissue in three studies; and urine in one study. Since EVs communicate with brain cells and quickly cross the brain barrier, they provide ample opportunity in SCZ to: explore and understand the complex pathophysiological changes that occur in brain cells, identify biological targets that can serve as diagnostic or prognostic markers of the disease, help in understanding the disease pathology, and facilitate the development of more safe and efficient drugs for therapy. The etiopathogenesis of SCZ is complex, involving an interplay of genetic, environmental, and biological factors that contribute to the development of the disease^48^. The included studies re-establish the role of these biological factors in disease pathogenesis. The identified signal markers were found to affect four major biological functions responsible for disease pathogenesis: 1) Modulation of structure and functions of neurotransmitters and their receptors, 2) Mitochondrial function, 3) Immune-related function, and 4) Metabolic pathway.

Certain biomolecules can affect more than one function, especially miRNAs, as each miRNA can target multiple mRNAs. MicroRNAs are short, non-coding RNAs (8-22 nucleotides) that lead to posttranslational blockade by targeting mRNA and subsequently regulating protein levels^49^. A total of 59 miRNAs were detected to be differentially expressed as potential signal markers, with some being upregulated and a few downregulated. Most of these miRNAs were found to play a role in the pathogenesis of SCZ^23,24,30–32^. The exact mechanism of their role in neuronal dysfunction in SCZ is complex due to their association with multiple genes. Their final effects are observed at the cellular surface (receptors) and intracellularly, affecting various pathways. They impact the structure and function of cell membrane receptors by targeting glutamate (miR-223 upregulation decreases *GRIN2B* and *GRIA2* receptor subunits)^31^, dopamine (downregulation of hsa-miR-486-5p, hsa-miR-199a- 3p, hsa-miR-144-5p, hsa-miR-451a, hsa-miR-143-3p, hsa-miR-142-3p)^24^, or interfere with multiple intracellular signaling pathways (GABA and BDNF signaling by miR-206 affecting neuronal development and survival, cognitive function and miR-132 synaptic plasticity)^32,50^. The neuronal network was also affected by them; miR-137 overexpression coupled with reduced COX6A2 levels in EVs was associated with impairment of parvalbumin interneurons (PVI) cortical microcircuit and cognitive dysfunction^35^. A few detected miRNAs were found to induce cell damage and neuronal death by affecting immune-related genes (miR-497, miR-675-3p associated with neuroinflammation and apoptosis)^51–56^ and inducing oxidative stress (upregulated miR-203A-3p increase oxidative stress by suppressing mRNA for DJ-1 protein known to be oxidative protective) (Fig. 2a, b)^23^.

Few differentially expressed miRNAs were found to affect synapse organization, neuron development, and cognitive deficits in schizophrenia (SCZ). Upregulation of has-miR-1246 and downregulation of hsa-miR-486-5p were associated with synaptic function. A few miRNAs were reported in the same study differentiating SCZ subgroups. Upregulated miRNAs (hsa-miR-1246, hsa-miR-5100, hsa-miR-3178, hsa-miR-7704, hsa-miR-203a-3p, hsa-miR-4521) and downregulated miRNAs (hsa-miR-451a) in cognitive deficit subset of SCZ targeted genes regulating neurogenesis and differentiation. Few of these dysregulated in TRS cases known to target genes implicated in neurodevelopmental functions. These molecular signals help in earlier detection of cognitive deficit and treatment-resistant SCZ cases^33^.

All the altered molecules were noted in SCZ cases except for 27 molecules where the differential expression was noted in the early psychosis patients, which include majority of proteins and a miRNA: miR-137, COX 6A2, LETM1, TRPM4, NCLX, CACNA- 1 C, ATP synthase activity, mitofusin 2 (MFN2), cyclophilin D (CYPD), syntaphilin (SNPH), humanin, MOTS-c, Complex I, Complex III decreased, GFAP, C3b opsonin, C5b-9, CD55, LIF, hippocalcin, Kalirin, Neurogranin, beta-adducin, ankyrin-2, Apolipoprotein A4 (APOA4), P0C671 Chromosome 6 open reading frame 222 (C6orf222) and Sorcin. Further cohort studies centered primarily on these signals will aid in early diagnosis and offer insights into etiopathogenesis^25,26,35,40–42^.

Long non-coding RNAs are longer than 200 nucleotides that do not translate into functional proteins. Numerous lncRNAs influence the transcription of neighbouring genes, regulating their expression and affecting mRNA functions, such as splicing, turnover, translation, and signaling pathways. As a result, lncRNAs influence several physiologically significant cellular processes, and changes in their distinct expression patterns may be employed as valuable disease signals^57^. Long-chain non-coding RNAs were differentially expressed in SCZ cases. MIAT was found to be overexpressed in drug naïve SCZ cases. MIAT (Gomafu) overexpression significantly decreased the expression of both *DISC1* and *ERBB4* genes, both of which are related to SCZ pathology, affecting neuronal excitatory transmission. However, the use of antipsychotic medication therapy, such as risperidone, restored the EV levels of MIAT in the aforementioned study, suggesting that medications can influence the EV levels of Gomafu downregulated PVT1 was associated with apoptosis, while its overexpression was found to act as a new oncogene^27,53,58,59^.

Another study highlighted the complex intricate network lncRNA-miRNA-mRNA(ceRNA) contributing to SCZ neuropathology. Hub genes EZH2 was associated with neural stem cell self-renewal and differentiation while, SIRT1 associated with self survival and apoptosis. MiR-26a-5p was associated to inflammation, lncRNA ENSG00000251562 linked to proliferation, growth and differentiation of CNS.The constructed ceRNA represented the intersection of neuroinflammation, dopaminergic signalling and apoptosis and was validated to differentiate first episode SCZ from healthy control^36^.

Circular RNAs (circRNAs) are RNAs with a unique circular structure generated through back-splicing processes^60^. Circular RNAs have been reported to modulate gene expression in the nucleus, acting as miRNA sponges (decoys) to prevent them from binding to their target mRNAs^61^. In SCZ, four validated circRNAs were found to be upregulated, targeting miR‐34a/c and miR‐499a, which are involved in SCZ pathogenesis. These miRNAs are involved in inflammation, stress response, MAPK signaling pathway, and protein processing in the endoplasmic reticulum^37^.

Altered protein signals found to be differentially expressed in EVs included GFAP, α-II-Spectrin, Aβ42, and specific mitochondrial and brain proteins. A study reported increased levels of GFAP and Aβ42, and reduced α-II-Spectrin levels compared to controls. GFAP is a known inflammatory marker of astrocytic pathology, and its elevation in SCZ is consistent with the immune system aberration theory in the disease pathology. On the other hand, reduced α-II-Spectrin, a cytoskeletal protein, indicates loss of neurons. The study further reported a positive association between psychopathology severity and α-II-Spectrin^38^. Similarly, Aβ42 protein was found to induce neuroinflammation by modulating the NFkB pathway and reduces neuronal viability by disturbing mitochondrial function. It was associated with cognitive dysfunction in Alzheimer’s disease^62–64^.

Complement and cytokine effector proteins showed differential expression in three studies. Raised complement mediators C3b and C5b-9 and reduced levels of C3 convertase inhibitor CD55 were responsible for neurotoxicity^42^. Similarly, a study reported differentially expressed complement activating proteins C3, C4A, C4B, C4BPA, C4BPB and PROS1. Also, using machine learning (XGBoost) developed Personalized Discrimination Score (PDS) based on the EV differential proteins for diagnosis and predict antipsychotic response to guide treatment decisions. These findings upheld immune dysregulation in SCZ pathogenesis^28^. Zhang et al., reported raised CD5 in SCZ compared to HC, increased CD33, CD40, CD36 in antipsychotic-resistant cases compared to non-antipsychotic-resistant cases. The study found DEPs associated with synaptic function (ITGA6 and ITGB3), vascular smooth muscle and endothelial functions (FN1 and PECAM1respectively), neural migration and intracellular calcium signalling (TENM2), dopamine release (EGFR) (Fig. 3)^29^.

A recent study reported, astrocyte-derived LIF, a key neuroprotective cytokine, was found at markedly reduced levels among patients with FEP^65^. Additionally, protein constituents of the mitochondrial oxidative phosphorylation system were differentially expressed in FEP patients compared to controls. As psychosis is one of the early manifestations of the disease, detection of candidate markers at this phase can identify patients early and facilitate the early initiation of therapy. In one study, reduced subunit proteins of mitochondrial oxidoreductases (complex I and Complex III subunits) were found to raise free radicals in SCZ, consistent with the oxidative stress hypothesis of SCZ pathogenesis^42,66,67^.

Mitochondrial-derived peptides (MDPs) are translated peptides originating from mitochondrial (mt) DNA genes that play a cytoprotective role in preserving mitochondrial function and cell viability under stressful conditions. A study by Goetzl in 2021 showed decreased exosomal levels of MOTS-c and Humanin proteins^41^. MOTS-c interacts with the nuclear genome, provides cytoprotection, inhibits inflammation, while humanin is an antiapoptotic peptide that interacts with Bcl-2-associated X protein of apoptosis (BAX), an apoptotic peptide that prevent apoptosis^68,69^. The study showed several other altered MDPs and their role in etiopathogenesis: overexpression of syntaphilin (SNPH), inducing N-methyl-D-aspartate (NMDA) excitotoxicity^70,71^, reduced MFN2 interfering with energy production and the accumulation of damaged mitochondria^72^, reduced levels of Cyclophilin D which regulates cell death in first episode psychosis^73^. Similarly, another study detected abnormal calcium ion channel proteins maintaining calcium homeostasis in mitochondria in FP. LETMI, TRPM4, and NCLX were reduced, and CACNA-1C was raised, affecting mitochondrial functions^40^. Changes in MDPs and imbalance in mitochondrial ionic homeostasis were shown to cause disturbances in bioenergetics, cell viability, and cell development.

Five brain proteins involved in glutamatergic synapses were found to be differentially expressed in psychotic patients. Hippocalcin and kalirin levels were increased, whereas neurogranin, beta-adducin, and ankyrin-2 were reduced^26^. Hippocalcin depresses synapses by recruiting AMPAR^74^, while kalirin enriched in the forebrain, plays a crucial role in morphological and functional plasticity at excitatory synapses and modulates the response to NMDAR activation^75–77^. Beta-adducin regulates dendritic spine stability through actin-based synapse formation and synapse stabilization^78^. Ankyrin-2, a member of the ankyrin family of proteins, maintains synapse stability^79^, and neurogranin increases synaptic strength^80^.

A study reported 127 unique proteins from the brain extracellular vesicles, distributed across prefrontal cortex (PFC), hippocampus (HC) and caudate brain regions in SCZ cases.Functional categorization region wise found affection of GABAergic synaptic transmission in prefrontal cortex and linking to neuronal impairment in other two regions. Also, exclusively in PFC region 19 EV proteomes were upregulated and 8 downregulated. These upregulated proteomes were found to be associated with structural and vesicular proteins (ACTA1, TUBA1A, TUBA1B, TUBA1C, TUBB4A, TUBB6, TUBB8B) responsible for movement, cell adhesion **(**NCAM1), stabilizing microtubule and formation and development of axons and dendrites (MAP1B, MAP4, MAP6, MAPT) and BSN, SYNPO responsible for synapse structure and functions, SYN1, SYN2, SNAP-25 regulate neurotransmitter while downregulated proteins were immunoglobulins. The above proteomic changes observed exclusively in PFC region represents the underlying multifaced disruption in synaptic structure and function, immune dysregulation, impaired neurotransmitter signalling and neuronal development (Fig. 3)^45,81^.

EVs secreted from intestinal epithelial cells and luminal bacteria contain differentially expressed three intestinal proteins. EV levels of ApoA4 was lower, whereas Bcl-2-interacting protein 5 (BNIP5 or C6orf222) and sorcin protein was higher^25^. Sorcin, an early marker of neurodegeneration was raised in psychotic patients^82^. Lesser is known about Bcl-2-interacting protein 5 (BNIP5 or C6orf222) and ApoA4 but reported to be associated with apoptosis, metabolism, and atherosclerosis^83–85^. These different proteins were associated with neuroprotection, neurodevelopment, metabolism, signaling, and apoptosis. These differentially expressed protein biomolecules lack validity and are limited by their small sample sizes. Nevertheless, these studies provide early changes of protein levels in the pathogenesis of SCZ, helping in diagnosis and understanding disease pathology.

Candidate molecules were identified due to underlying metabolic disturbances in SCZ, specifically impaired insulin signaling pathways. Compared to controls, drug naïve first episode SCZ cases showed reduced levels of insulin signal transduction protein pS312-IRS-1. This signal was detected in first-episode and drug-free states, eliminating the influence of drugs as a confounder. Similarly, another study demonstrated a trend of lower insulin signalling molecules in blood EVs of neuronal origin^48,50^.

Serum-derived EVs demonstrated significant differences in estimated levels of 25 metabolites in SCZ compared to controls. Ten of them were upregulated and 15 downregulated. All candidate biomolecules were validated in separate cohorts. These metabolites showed excellent performance in differentiating between patients and controls in three test sets of participants. Validity was confirmed through multi-correlation coefficient analysis, assessing the relationship between the 25 metabolites and potential confounders such as age, disease severity, and dysfunction in glycerophospholipid metabolism. Additionally, these 25 metabolites were significantly associated with the Positive and Negative Syndrome Scale (PANSS) negative score in the second and third test participants. The study also identified six metabolites differentiating drug naïve/drug-free SCZ group from chronically treated cases^46^. Similarly, another study reported seven differential lipid molecules belonging to glycerophospholipids and sphingolipids, Cer(d18:0/24:1), Cer(d18:1/18:0), Cer(d18:1/24:1), PC(16:0_16:1), PE(16:0_20:4), PE(16:0_22:5), and SM(d18:0/18:0).These lipids mainly the SM are components of cell membrane and signalling molecules associated with first and/or second messengers to regulate signal transduction processes cell growth, differentiation, senescence, and apoptosis (Fig. 4)^47^.

### Strength and limitations

Our review has certain limitations, such as using only English-language literature, excluding trial registries, and not conducting a grey literature search.

- Although our study finds the biomolecules that express differently in SCZ, further research in larger cohorts is needed before clinical benefits can be realized. Most of the studies till date were conducted in small samples and hence lacks validity. Designing and conducting studies with larger sample sizes improves statistical power and generalizability.
- It is noteworthy that most of the evidence emerged from exosomes was from cross-sectional studies and not longitudinal studies. Hence, these differentially expressed candidate markers cannot be considered as causative factors in the development of disease. Major challenge in the follow-up studies is patient retention in the study particularly involving psychiatric illness. Consistent engagement and regular communication with patient and patient party is required to reduce high attrition rates.
- Most of the studies have participants taking antipsychotic medications and few are drug naïve or drug free. This can add to the list confounders. Also factors like socioeconomic status, comorbidities, smoking, alcohol, drug abuse, and details regarding the selection of controls were missing. Designing randomized clinical studies can minimize biases and better evaluate the effects of potential biomarkers.
- Few studies have included other psychiatric cases (e.g. bipolar disorder) along with SCZ, hence observed differentially expressed markers may not be specific to SCZ. Expand studies to include a broader range of psychiatric disorders as comparators to improve the specificity of identified biomarkers for schizophrenia.
- On the analytical aspect there are no universal standard protocol for EV isolation and EV enrichment specifically to CNS. This will further strengthen the uniform methods and facilitate the reproducibility and comparability of findings. Furthermore, apply a combination of separation methods such as size exclusion chromatography, density gradient ultracentrifugation, and asymmetric flow field-flow fractionation (AF4) to improve the purity and specificity of EV populations.

Despite these inadequacies, the study provides the relevant knowledge needed to support clinical studies investigating the role of EVs in diagnosing and treating SCZ.

- There has been an influx of research works in SCZ. This scoping review incorporates recent advancements of newly published studies in EVs field, ensuring up-to-date and comprehensive synthesis of evidence.
- This scoping review identifies key research gaps in the identification of differential biomolecules in schizophrenia underscoring the need for large population studies, longitudinal design, and exploring population-specific, disease-specific differential biomolecular detection and validation essential for the advancement in the field.
- The study summarizes potential signal markers both in the early and late stage of the disease encompassing differential genetic, proteomic, metabolomic molecular signals and their functional links. The study not only lays the groundwork for further research but also makes it easier to comprehend the neuropathology of the condition.

## Conclusion

Identifying differential biomolecules in schizophrenia is essential to complement clinical diagnosis. Screening of studies have identified 83 potential altered molecular signals in EVs isolated from different biological tissues in SCZ patients, comprising 38 metabolites, 22 miRNAs, 15 proteins, 4 circRNAs, 2 lncRNAs and 2 ceRNAs. This comprehensive scoping review identifies the potential of EVs in diagnostics and therapeutics in SCZ and explored the role of EVs in disease neuropathogenesis. The findings in the study will help in designing advanced clinical studies to facilitate its clinical utility through large scale studies.

## Supplementary information

Supplementary tables S1, S2, S3, S5, S6

Supplementary table S4

## Data Availability

There is no primary data generated in the current study.
