# Supplementary material for: Potential of extracellular vesicle cargo as molecular signals in Schizophrenia: a scoping review
Source: Schizophrenia (Heidelb). 2025 Feb 12;11(1):17. doi: 10.1038/s41537-025-00566-5 (PMC11822128; doi:10.1038/s41537-025-00566-5)
Supplement: Supplementary file 1 — Supplementary tables S1, S2, S3, S5, S6 [file 41537_2025_566_MOESM1_ESM.docx]

**Supplementary Material**

**Supplementary Table S1:** Extracellular Vesicle (EV) isolation methods

| **Method** | **Procedure** | **Advantage** | **Disadvantage** |
| --- | --- | --- | --- |
| **Ultracentrifugation** | | | |
| Differential ultracentrifugation^1-3^ | Differential centrifugation works on a very basic principle: larger and denser particles sediment out first. This process allows for the sequential separation of distinct extracellular components of a fluidic sample according to density, size, and shape under specific centrifugal forces. Before isolating purified exosomes, a cleaning step involving low-speed centrifugation (e.g., 300 ×g) to remove large bioparticles may be carried out. This is followed by multiple cycles of centrifugation with centrifugal force ranging from 2000 ×g to 100,000 ×g, which remove contaminants like cell derbies, apoptotic bodies, and protein aggregates in that order. | Little or no need for pretreatment of samples, little technical expertise,  applicable for large volume processing. | Low purity,  Cost of equipment, long isolation times, damage of EVs due to centrifugation at high speed is common, high working volume. |
| Isopycnic type of density-gradient  ultracentrifugation for high-quality exosome isolation^3-5^ | Layers of biocompatible medium covering the range of particle densities in the sample are put into a tube, with progressively lower densities from bottom to top (e.g., iodoxinol or sucrose). Sample is added to the top of the density gradient medium, centrifuged for 16hr (100,000 ×g). The extracellular elements eventually settle into an isopycnic position—a static location inside the layer of the same density—along with exosomes, apoptotic bodies, and protein clumps. This technique makes it simple to separate components with varying buoyant densities; exosomes stay in the layer of medium between 1.10 and 1.18 g, whereas protein clumps concentrate at the bottom of the centrifuge tube. | Pure isolates and higher yield, little or no need for pretreatment of samples. | Low efficiency, complexity, exosomal  aggregation, viral particles migrate to the same density gradient with the EVs, labour -intensive, time consuming, not suitable for high throughput applications, Cost of equipment, long isolation times, damage of EVs due to centrifugation at high speed,  require trained technicians, low portability. |
| Rate-Zonal centrifugation or Moving-Zone density-gradient  ultracentrifugation ^3-5^ | The widely utilized isopycnic ultracentrifugation relies solely on the variations in density. Though effective in separating common contaminants like protein aggregates, other extracellular vesicles, including microvesicles, that have a buoyant density comparable to exosomes but a different size, cannot be separated in the same way. The moving-zone ultracentrifugation (rate zonal centrifugation) process uses a medium whose density is less than the total solute concentration in the sample. After centrifugation, all of the solutes in the sample will be sequentially separated based on mass/size and density since the solutes' densities are greater than the gradient medium's. This will enable the isolation of vesicles with similar densities but different sizes. | Pure isolates and higher yield, little or no need for pretreatment of samples. | Low efficiency, complexity, exosomal  Aggregation labour -intensive, time consuming, not suitable for high throughput applications, Cost of equipment, long isolation times, damage of EVs due to centrifugation at high speed, require trained technicians. |
| **Size Exclusion- based EV isolation- separation of EVs based on size** | | | |
| Ultrafiltration or microfiltration^3-5^ | **Two types of ultrafiltration devices**:  **Two tandem-configured microfilters-** With size-exclusion limits between 20 and 200 nm. Large vesicles, such as apoptotic bodies, and most microvesicles are retained in the 200 nm membrane when they pass through the two membranes. Smaller particles, like as proteins, pass through the 20 nm microfilter, while vesicles with a diameter of 20 to 200 nm stay on the bottom.    **Sequential ultrafiltration-**  Extracellular fluids are filtered through a 1000-nm filter in order to remove big particles such as apoptotic bodies, cells, and their debris. Subsequently, a 500-kD MWCO second filter is used to filter out free proteins and other tiny particles from the filtrate. Using a 200-nm filter, exosomes with diameters ranging from 50 to 200 nm can be extracted from the filtrate. Example: "ExoMirTM exosome isolation" kit. | Large volume processing;  Rapid isolation, Simple and portable, low cost(table) | Vesicle clogging and entrapment is one of the most noticeable concerns; this can shorten the lifespan of the expensive membranes and result in a low separation yield.  If transmembrane pressure is applied incorrectly during ultrafiltration, it may negatively impact the separated exosomes' natural condition and cause them to lose their functionality. |
| Tangential flow filtration^3-4^ | The feed stream flows parallel to the membrane. Only a portion of the flow crosses the membrane when pressure is applied to the flow stream, owing to the adjustments made to the hydrodynamic flow force. During the tangential flow filtration process, the retentate can then be recycled back to the feed reservoir for more filtration. | Pure, scalable, reproducible, gentle on the integrity  of EVs, efficient, membrane is continuously subjected to a parallel flow force, which effectively reduces clogging (via constant flushing).  High yield results from recirculating retainate. | Complex setup. |
| Size-exclusion chromatography (SEC)^3-5^ | Sample is introduced atop a column that has been filled with a matrix with defined pore sizes. Smaller particles (smaller than the matrix pore size) are kept longer and elute mostly in later fractions, while bigger particles, driven by gravity or pump pressure, pass through the matrix fast and without entering the pores and collected as early fractions. | SEC produce a high yield isolation and high purity while preserving biophysical and functional properties of the isolated vesicles, low cost and  short isolation time, used for both small and large sample capacity. | Long running time,  contaminants like protein aggregates and lipoproteins hence advisable to combine with ultrafiltration, additional requirement for exosome enrichment. |
| Asymmetrical flow field-flow fractionation (AF4) systems^2,5,6^ | This procedure involves injecting and pushing the sample along the chamber's length by parabolic flow. A crossflow which is perpendicular to the parametric flow is employed concurrently to separate the particles. Larger particles are driven closer to the chamber walls, where the parabolic flow is slower, (more impacted by the crossflow) and the smaller particles which are less impacted by the crossflow stay in the center of the parabolic flow. The smaller particles elute earlier than the bigger particles. | requires a smaller volume of starting material than in conventional chromatography and is able to produce EVs of high purity. | AF4 is not specific to EVs in its standard configuration. |
| **Immunoaffinity based EV capture-** | | | |
| Immunoaffinity capture-based EV isolation methods^3-5^ | The basis of immunoaffinity-based isolation is: certain proteins and receptors are present in all exosomes and the specific binding between these protein markers and their corresponding antibodies can separate exosomes. Example:  Exosome isolation and analysis kit (Abcam), Exosome-human CD63 isolation reagent (Thermofisher) and Exosome Isolation Kit CD81/CD63 (Miltenyi Biotec).    **Solid matrices for antibody immobilization**  Antibodies are immobilized to a solid surface for exosome separation.  Matrices such as plates, beads, chromatography, and different kinds of microfluidic devices were employed. The most often employed of them have been submicron-sized magnetic particles, which are routinely used for immuno-precipitation of recombinant proteins.  Microplate based;Magnetic beads(Tim4 protein immobilized onto beads; EpCAM antibody-coated magnetic beads);Chemical antibody-based next gen immunoaffinity are the other methods of immunoaffinity approach | Technique can easily complement other isolation methods, while offering increased efficiency, specificity and integrity in the recovery of EVs from complex and viscous  fluids.  Antibody-  coated magnetic bead system allowed the isolation of specific  subpopulation of exosomes. Example: EpCAM | Rigorous definition of specific EV markers remains unclear,  antibody availability  and the presence of these markers in the whole population negatively affects it, biomarkers should be expressed on the surface of the EVs and should also be fully membrane-bound.  High cost of antibodies and non-neutral pH and nonphysiological elution buffers used to separate exosome from antibodies could affect the biological functions of exosomes,  Low processing volume and yields. |
| **Microfluidic technique for exosome isolation** | | | |
| Immunoaffinity-based microfluidics^1,3^ | The immunoaffinity-microfluidic-based exosome separation devices work on the principle of corresponding antibodies mounted on the chips specifically recognize exosome markers.  Example: Exochip.  Exosomes are directly captured on the surface of the microfluidic devices which are functionalized with capture agents or beads functionalized with exosome specific antibodies are pre-incubated with exosome-containing serum, followed by processing with microfluid device for assessing the expression levels of specific cancer-associated markers. | Fast isolation from small quantity of sample. Allows real-time on-chip exosome analysis.  High purity and cost effective, portable as it is miniaturized apparatus, easily automated and integrated with diagnosis. | Limited by isolation of only  specific subset of exosomes, high cost, and difficulty in maintaining the natural structure of exosomes…  Low sample capacity….  Sample loss during elution process,  Difficult to detach exosomes for the further application. |
| Electrical based separation^1,3^ | Uses the dielectrophoresis (DEP) force which helps transport the dielectric particles polarized in an irregular electric field. DEP force leads to the migration of electrically polarizable particles and the force applied to the particles depends on the particle size, applied field frequency, medium viscosity. Exosomes are separated based on the difference in DEP force applied to the particles (size-dependent). | Applicable to process small sample volume  High purity  Fast processing  High sensitivity | Complicated system  _Multiple experiments required to  establish the optimal condition to  enrich exosome sized EVs.  Possibility of forming gas bubble.  Heating of the solution. |
| Hydrodynamic Properties based separation^1,3^ | Particle separation is aided by a fluid mixture having viscoelastic characteristics. PEO, a polymer that is added to a fluid to increase its viscoelasticity. It creates elastic lift forces in the fluid that have the ability to move particles in particular directions. There are two inlets and three outputs on the microdevice. There is a long, narrow passage inside the device. One inlet is used to introduce sample fluid, and the other is used to sheath fluid containing PEO. Particles of varying sizes are pushed to different locations in the channel by the elastic lift forces produced by the PEO as the fluids mix. Larger particles go faster toward the channel's center and leave through the center outlet. Smaller particles gather through the side exits as they travel more slowly. | Applicable to process small sample volume  High purity  Simple channel structure | Comparably long processing time  Potential risk of clogging. |
| Acoustic field based separation^1,3^ | Utilizes acoustic radiation forces applied to the particles inside the medium. The size and density of particles have a significant impact on the acoustic radiation force. By integrating with microfluidic platforms, EVs are separated from the larger particles based on the difference in the radiation force applied to the particles. | Fast processing  High separation yield  Applicable to process small sample volume | Low isolation throughput  Complicated system  Complex fabrication process  Heavy benchtop instrumentation  needed for the generation of acoustic wave |
| **Precipitation** |  |  |  |
| PEG^3^ | Polyethylene glycol (PEG) a polymer with molecular weights between 6000 and 20000 Da is used. Large contaminating particles like cell debris and apoptotic bodies must first be removed by pretreatment. The pre-treated samples must then be incubated with PEG solution at 4°C for an entire night. Subsequently, the exosomes that precipitate are extracted using a low-speed centrifugation method (1500 ×g). | Have been proposed as inexpensive and quick protocols, easy, donot require equipments, requiring little technical expertise or expensive equipment**.**  •Suitable for both small and large sample  volume  • High efficiency  High yield | Preisolation and postisolation steps aimed at minimizing contamination.  Requires sample pretreatment like ultracentrifugation and or filtration,  • Extended processing time  • Require complicated clean-up steps  • Affecting downstream analysis and quantification |

**Reference**

1. Kumar, K., Kim, E., Alhammadi, M., Reddicherla, U., Aliya, S., Tiwari, J. N. et al. Recent millers in microfluidic approaches for the isolation and detection of exosomes. TrAC - *Trends Anal. Chem.* **159**, 116912 (2023). https://doi.org/10.1016/j.trac.2022.116912

2. Busatto, S., Vilanilam, G., Ticer, T., Lin, W. L., Dickson, D. W., Shapiro, S.et al. Tangential flow filtration for highly efficient concentration of extracellular vesicles from large volumes of fluid. *Cells* **7**, 273 (2018). https://doi.org/10.3390/cells7120273

3. Yang, D., Zhang, W., Zhang, H., Zhang, F., Chen, L., Ma, L., et al. Progress, opportunity, and perspective on exosome isolation - Efforts for efficient exosome-based theranostics. *Theranostics* **10**, 3684–707 (2020). https://doi.org/10.7150/thno.41580.

4. Kırbaş, O. K., Bozkurt, B. T., Asutay, A. B., Mat, B., Ozdemir, B., Öztürkoğlu, D., et al. Optimized Isolation of Extracellular Vesicles From Various Organic Sources Using Aqueous Two-Phase System. Sci Rep. **9,** 1–11 (2019). https://doi.org/10.1038/s41598-019-55477-0

5. De Sousa, K. P., Rossi, I., Abdullahi, M., Ramirez, M,I., Stratton, D., & Inal, J. M. Isolation and characterization of extracellular vesicles and future directions in diagnosis and therapy. *Wiley Interdiscip. Rev. Nanomed. Nanobiotechnol.* **15**, 1–29 (2023). https://doi.org/10.1002/wnan.1835

6. Welsh, J. A., Goberdhan, D. C. I., O'Driscoll, L., Buzas, E. I., Blenkiron, C., Bussolati, B., et al. Minimal information for studies of

extracellular vesicles (MISEV2023): From basic to advanced approaches. *J. Extracell. Vesicles* **13,** e12404 (2024).

https://doi.org/10.1002/jev2.12404.

**Supplementary Table S2:** **Preferred Reporting Items for Systematic reviews and Meta-Analyses extension for Scoping Reviews (PRISMA-ScR) Checklist**

| **ECTION** | **ITEM** | **PRISMA-ScR CHECKLIST ITEM** | **REPORTED ON PAGE #** |
| --- | --- | --- | --- |
| **TITLE** | | | |
| Title | 1 | Identify the report as a scoping review. | 1 |
| **ABSTRACT** | | | |
| Structured summary | 2 | Provide a structured summary that includes (as applicable): background, objectives, eligibility criteria, sources of evidence, charting methods, results, and conclusions that relate to the review questions and objectives. | 2 |
| **INTRODUCTION** | | | |
| Rationale | 3 | Describe the rationale for the review in the context of what is already known. Explain why the review questions/objectives lend themselves to a scoping review approach. | 3-4 |
| Objectives | 4 | Provide an explicit statement of the questions and objectives being addressed with reference to their key elements (e.g., population or participants, concepts, and context) or other relevant key elements used to conceptualize the review questions and/or objectives. | 4 |
| **METHODS** | | | |
| Protocol and registration | 5 | Indicate whether a review protocol exists; state if and where it can be accessed (e.g., a Web address); and if available, provide registration information, including the registration number. | NA |
| Eligibility criteria | 6 | Specify characteristics of the sources of evidence used as eligibility criteria (e.g., years considered, language, and publication status), and provide a rationale. | 5 |
| Information sources* | 7 | Describe all information sources in the search (e.g., databases with dates of coverage and contact with authors to identify additional sources), as well as the date the most recent search was executed. | 5 |
| Search | 8 | Present the full electronic search strategy for at least 1 database, including any limits used, such that it could be repeated. | Table S2, S7-8 |
| Selection of sources of evidence† | 9 | State the process for selecting sources of evidence (i.e., screening and eligibility) included in the scoping review. | 4-5 |
| Data charting process‡ | 10 | Describe the methods of charting data from the included sources of evidence (e.g., calibrated forms or forms that have been tested by the team before their use, and whether data charting was done independently or in duplicate) and any processes for obtaining and confirming data from investigators. | 5 |
| Data items | 11 | List and define all variables for which data were sought and any assumptions and simplifications made. | 5 |
| Critical appraisal of individual sources of evidence§ | 12 | If done, provide a rationale for conducting a critical appraisal of included sources of evidence; describe the methods used and how this information was used in any data synthesis (if appropriate). | NA |
| Synthesis of results | 13 | Describe the methods of handling and summarizing the data that were charted. | 5-6 |
| **RESULTS** | | | |
| Selection of sources of evidence | 14 | Give numbers of sources of evidence screened, assessed for eligibility, and included in the review, with reasons for exclusions at each stage, ideally using a flow diagram. | 6 & Table S4 |
| Characteristics of sources of evidence | 15 | For each source of evidence, present characteristics for which data were charted and provide the citations. | 6-24 & Table S4 |
| Critical appraisal within sources of evidence | 16 | If done, present data on critical appraisal of included sources of evidence (see item 12). | NA |
| Results of individual sources of evidence | 17 | For each included source of evidence, present the relevant data that were charted that relate to the review questions and objectives. | 6-24 |
| Synthesis of results | 18 | Summarize and/or present the charting results as they relate to the review questions and objectives. | 6-24 |
| **DISCUSSION** | | | |
| Summary of evidence | 19 | Summarize the main results (including an overview of concepts, themes, and types of evidence available), link to the review questions and objectives, and consider the relevance to key groups. | 25-28 |
| Limitations | 20 | Discuss the limitations of the scoping review process. | 29 |
| Conclusions | 21 | Provide a general interpretation of the results with respect to the review questions and objectives, as well as potential implications and/or next steps. | 29 |
| **FUNDING** | | | |
| Funding | 22 | Describe sources of funding for the included sources of evidence, as well as sources of funding for the scoping review. Describe the role of the funders of the scoping review. | Page S18-S23 (Table S6) |

JBI = Joanna Briggs Institute; PRISMA-ScR = Preferred Reporting Items for Systematic reviews and Meta-Analyses extension for Scoping Reviews.

* Where *sources of evidence* (see second footnote) are compiled from, such as bibliographic databases, social media platforms, and Web sites.

† A more inclusive/heterogeneous term used to account for the different types of evidence or data sources (e.g., quantitative and/or qualitative research, expert opinion, and policy documents) that may be eligible in a scoping review as opposed to only studies. This is not to be confused with *information sources* (see first footnote).

‡ The frameworks by Arksey and O’Malley (6) and Levac and colleagues (7) and the JBI guidance (4, 5) refer to the process of data extraction in a scoping review as data charting*.*

§ The process of systematically examining research evidence to assess its validity, results, and relevance before using it to inform a decision. This term is used for items 12 and 19 instead of "risk of bias" (which is more applicable to systematic reviews of interventions) to include and acknowledge the various sources of evidence that may be used in a scoping review (e.g., quantitative and/or qualitative research, expert opinion, and policy document).

*From:* Tricco AC, Lillie E, Zarin W, O'Brien KK, Colquhoun H, Levac D, et al. PRISMA Extension for Scoping Reviews (PRISMAScR): Checklist and Explanation. Ann Intern Med. 2018;169:467–473. [doi: 10.7326/M18-0850](http://annals.org/aim/fullarticle/2700389/prisma-extension-scoping-reviews-prisma-scr-checklist-explanation)

**Supplementary Table S3:** Search Strategy for Databases

| **S.no** | **Database** | **Search Strategy** | **Hits** |
| --- | --- | --- | --- |
| 1 | PubMed (NCBI) | (("Extracellular Vesicles"[MeSH Terms] OR "exosome*"[Text Word] OR "extracellular vesicle*"[Text Word] OR "exovesicle*"[Text Word] OR "apoptotic body"[Text Word] OR "apoptotic bodies"[Text Word] OR "EVs"[Text Word] OR "ectosome*"[Text Word] OR "cell derived microparticle*"[Text Word] OR "shedding microvesicle*"[Text Word] OR "shedding microvesicle*"[Text Word] OR "cell membrane microparticle*"[Text Word])) AND (((("Schizophrenia"[MeSH Terms] OR "Schizophrenia"[Text Word] OR "Schizotypal Personality Disorder"[MeSH Terms] OR "Schizotypal Personality Disorder"[All Fields] OR "schizophrenia, treatment resistant"[MeSH Terms] OR "Schizophrenia Spectrum and Other Psychotic Disorders"[MeSH Terms] OR "Schizophrenia Spectrum and Other Psychotic Disorders"[Text Word]) OR "schizophrenia, paranoid"[MeSH Terms]) OR "schizophrenia paranoid"[All Fields] OR "schizophrenia, disorganized"[MeSH Terms] OR "schizophrenia disorganized"[Text Word] OR "schizophrenia, childhood"[MeSH Terms] OR "schizophrenia childhood"[Text Word] OR "schizophrenia, catatonic"[MeSH Terms] OR "schizophrenia catatonic"[Text Word]))  Filters: Language: English | 73 |
| 2 | EMBASE (Elsevier) | (('Extracellular Vesicles'/exp OR exosome* OR 'extracellular vesicle*' OR exovesicle* OR 'apoptotic body' OR 'apoptotic bodies' OR EVs OR ectosome* OR 'cell derived microparticle*' OR 'shedding microvesicle*' OR 'shedding microvesicle*' OR 'cell membrane microparticle*')) AND ((((Schizophrenia/exp OR Schizophrenia OR 'Schizotypal Personality Disorder'/exp OR 'Schizotypal Personality Disorder' OR 'schizophrenia, treatment resistant'/exp OR 'Schizophrenia Spectrum and Other Psychotic Disorders'/exp OR 'Schizophrenia Spectrum and Other Psychotic Disorders') OR 'schizophrenia, paranoid'/exp) OR 'schizophrenia paranoid' OR 'schizophrenia, disorganized'/exp OR 'schizophrenia disorganized' OR 'schizophrenia, childhood'/exp OR 'schizophrenia childhood' OR 'schizophrenia, catatonic'/exp OR 'schizophrenia catatonic'))  Filters: Language: English | 208 |
| 3 | CINAHL (EBSCHO) | (((MH "Extracellular Vesicles+") OR exosome* OR "extracellular vesicle*" OR exovesicle* OR "apoptotic body" OR "apoptotic bodies" OR EVs OR ectosome* OR "cell derived microparticle*" OR "shedding microvesicle*" OR "shedding microvesicle*" OR "cell membrane microparticle*")) AND (((((MH Schizophrenia+) OR Schizophrenia OR (MH "Schizotypal Personality Disorder+") OR "Schizotypal Personality Disorder" OR (MH "schizophrenia, treatment resistant+") OR (MH "Schizophrenia Spectrum and Other Psychotic Disorders+") OR "Schizophrenia Spectrum and Other Psychotic Disorders") OR (MH "schizophrenia, paranoid+")) OR "schizophrenia paranoid" OR (MH "schizophrenia, disorganized+") OR "schizophrenia disorganized" OR (MH "schizophrenia, childhood+") OR "schizophrenia childhood" OR (MH "schizophrenia, catatonic+") OR "schizophrenia catatonic"))  Filters: Language: English | 12 |
| 4 | Scopus (Elsevier) | ( ( INDEXTERMS ( "Extracellular Vesicles" ) OR TITLE-ABS-KEY ( exosome* ) OR TITLE-ABS-KEY ( "extracellular vesicle*" ) OR TITLE-ABS-KEY ( exovesicle* ) OR TITLE-ABS-KEY ( "apoptotic body" ) OR TITLE-ABS-KEY ( "apoptotic bodies" ) OR TITLE-ABS-KEY ( evs ) OR TITLE-ABS-KEY ( ectosome* ) OR TITLE-ABS-KEY ( "cell derived microparticle*" ) OR TITLE-ABS-KEY ( "shedding microvesicle*" ) OR TITLE-ABS-KEY ( "shedding microvesicle*" ) OR TITLE-ABS-KEY ( "cell membrane microparticle*" ) ) ) AND ( ( ( ( INDEXTERMS ( schizophrenia ) OR TITLE-ABS-KEY ( schizophrenia ) OR INDEXTERMS ( "Schizotypal Personality Disorder" ) OR ALL ( "Schizotypal Personality Disorder" ) OR INDEXTERMS ( "schizophrenia, treatment resistant" ) OR INDEXTERMS ( "Schizophrenia Spectrum and Other Psychotic Disorders" ) OR TITLE-ABS-KEY ( "Schizophrenia Spectrum and Other Psychotic Disorders" ) ) OR INDEXTERMS ( "schizophrenia, paranoid" ) ) OR ALL ( "schizophrenia paranoid" ) OR INDEXTERMS ( "schizophrenia, disorganized" ) OR TITLE-ABS-KEY ( "schizophrenia disorganized" ) OR INDEXTERMS ( "schizophrenia, childhood" ) OR TITLE-ABS-KEY ( "schizophrenia childhood" ) OR INDEXTERMS ( "schizophrenia, catatonic" ) OR TITLE-ABS-KEY ( "schizophrenia catatonic" ) ) )  Filters: Language: English | 176 |

**Supplementary Table S5:** Excluded articles at the full text stage with justification

| **Sl No** | **Study ID** | **Reasons for exclusion** | **Reference** |
| --- | --- | --- | --- |
| 1 | Tsilioni 2014 | Review | Tsilioni,I., Panagiotidou, S., & Theoharides, T.C. Exosomes in neurologic and psychiatric disorders. *Clin. Ther.* **36,** 882–888 (2014). https://doi.org/10.1016/j.clinthera.2014.05.005 |
| 2 | Desmeules 2023 | Poster | Desmeules, C., Bechard, L., Huot-Lavoie, M., Corbeil, O., Essiambre, A. M., Anderson E., et al. Psychosis and trauma - Measurements of blood-based exosomal biomarkers from brain origins: Preliminary results. *Early Interv. Psychiatry* **17**, 306 (2023). https://doi.org/10.1111/eip.13409. |
| 3 | Martinez 2024 | Review | Martinez, B., & Peplow, P.V. MicroRNAs as potential biomarkers for diagnosis of schizophrenia and influence of antipsychotic treatment. *Neural Regen Res*. **19,** 1523–1531 (2024). https://doi.org/10.4103/1673-5374.387966 |
| 4 | Do 2023 | Commentary | Do, K. Q. Bridging the gaps towards precision psychiatry: Mechanistic biomarkers for early detection and intervention. *Psychiatry Res*. **321,** 115064 (2023). https://doi.org/10.1016/j.psychres.2023.115064 |
| 5 | Guo 2024 | Review | Guo, C., Bai, Y., Li, P., & He, K. The emerging roles of microbiota-derived extracellular vesicles in psychiatric disorders. *Front. Microbiol*. **15,** 1383199 (2024). https://doi.org/10.3389/fmicb.2024.1383199 |
| 6 | Zhang 2024 | Review | Zhang, R., Lei, X., Ren, J., & Zhang, C. The roles of extracellular vesicle-derived microRNAs in schizophrenia: A scoping review. *Schizophr. Res*. **270**, 162–164 (2024). https://doi.org/10.1016/j.schres.2024.04.011 |
| 7 | Ansarey 2021 | Review | Ansarey, S. H. Inflammation and JNK's Role in Niacin-GPR109. A diminished flushed effect in microglial and neuronal cells with relevance to schizophrenia. *Front.* *Psychiatry* **12**, 771144 (2021). https://doi.org/10.3389/fpsyt.2021.771144 |
| 8 | Zhang 2021 | Review | Zhang, N., He, F., Li, T., Chen, J., Jiang, L., Ouyang, X.P., & Zuo, L. Role of exosomes in brain diseases*. Front. Cell Neurosci.* **15**, 743353 (2021). https://doi.org/10.3389/fncel.2021.743353 |
| 9 | Munir 2020 | Review | Munir, J., Yoon, J. K., & Ryu, S. Therapeutic miRNA-enriched extracellular vesicles: Current approaches and future prospects. *Cells* **9,** 2271 (2020). https://doi.org/10.3390/cells9102271 |
| 10 | Fu 2023 | Review | Fu, L., & Zhang, C. Advances in the role of circular RNA in schizophrenia. *J. Shanghai Jiao Tong Univ. (Med. Sci.)* **43**, 1445–1449 (2023). |
| 11 | Tunset 2019 | Conference abstract | Tunset, M., Haslene-Hox, H., Vaaler, A., Sulheim, E., Kondziella, D. P. 393 Changes in peripheral blood extracellular vesicles in psychotic patients determined by vesicle characterization and proteomics**.** *Eur. Neuropsychopharmacol.* **29**, S278–S279 (2019). https://doi.org/10.1016/j.euroneuro.2019.09.407 |
| 12 | Gruzdev 2019 | Review | Gruzdev, S. K., Yakovlev, A. A., Druzhkova, T. A., Guekht, A. B., & Gulyaeva, N. V. The missing link: How exosomes and miRNAs can help in bridging psychiatry and molecular biology in the context of depression, bipolar disorder and schizophrenia. *Cell Mol. Neurobiol.* **39**, 729–750 (2019). https://doi.org/10.1007/s10571-019-00684-6 |
| 13 | Xavier 2019 | Conference abstract F.131 | [Xavier, G., Santoro, M., Ota, V., Talarico, F., Spindola, L., Oliveira, G. et al. F131. Evaluation of extracellular vesicles miRNA differential expression among first episode psychosis. *Eur. Neuropsychopharmacol* **29**, S1181–S1182 (2019). https://doi.org/10.1016/j.euroneuro.2018.08.211](Xavier,%20G.,%20Santoro,%20M.,%20Ota,%20V.,%20Talarico,%20F.,%20Spindola,%20L.,%20Oliveira,%20G.%20et%20al.%20F131%20Evaluation%20of%20extracellular%20vesicles%20miRNA%20differential%20expression%20among%20first%20episode%20psychosis.%20European%20Neuropsychopharmacology 29,%20S1181-S1182(2019).%20https://doi:%2010.1016/j.euroneuro.2018.08.211) |
| 14 | Xavier 2019 | Conference abstract M95 | Xavier, G., Talarico, F., Santoro, M., Ota, V., Moretti, P., Costa, G. et al. Schizophrenia related miRNAs selection in a brazilian sample. *Eur. Neuropsychopharmacol.* **29**, S1007 (2019). https://doi.org/10.1016/j.euroneuro.2017.08.402 |
| 15 | Li 2019 | Review | Li, L. & Wang, J. Roles of extracellular microRNAs in central nervous system. *ExRNA 1*, **13** (2019). https://link.gale.com/apps/doc/A603082654/AONE?u=anon~ec328bfa&sid=googleScholar&xid=79210568 |
| 16 | Al-Amin 2019 | Conference abstract | Al-Amin, H., El-Asrag, M., Ghuloum, S., Cardno, A., Chamali, R., Kiwan, N. et al. Screening for major risk alleles for Schizophrenia in Qatari population. *Behav. Genet.* **49**, 534 (2019). |
| 17 | Ashley 2018 | Invitro-animal | Ashley, J., Cordy, B., Lucia, D., Fradkin, L.G., Budnik, V., Thomson, T. Retrovirus-like Gag Protein Arc1 Binds RNA and Traffics across Synaptic Boutons. *Cell*. **172**, 262–274 (2018). https://doi.org/10.1016/j.cell.2017.12.022 |
| 18 | Schneider 2016 | Conference abstract_ | Schneider, E. M., Strunz, M., Lowe, R., & Bechter, K. Characterization of microparticles derived from cultured macrophages and cerebrospinal fluid of patients with schizophrenic and affective disorder. [*Neurology, Psychiatry and Brain Research*](https://www.sciencedirect.com/journal/neurology-psychiatry-and-brain-research) 22, 19 (2016). https://doi.org/[10.1016/j.npbr.2015.12.047](http://dx.doi.org/10.1016/j.npbr.2015.12.047) |
| 19 | Zhu 2015 | Invitro-animal | Zhu, C. Y., Shen, Y., & Xu, Q. Propagation of dysbindin-1B aggregates: exosome-mediated transmission of neurotoxic deposits. *Neuroscience* **291,**301-16(2015). https://doi.org/10.1016/j.neuroscience.2015.02.016 |
| 20 | De Toro 2015 | Review | De Toro, J., Herschlik, L., Waldner, C., & Mongini, C. Emerging roles of exosomes in normal and pathological conditions: new insights for diagnosis and therapeutic applications. *Front. Immunol*. **6**, 203 (2015). https://doi.org/10.3389/fimmu.2015.00203. |
| 21 | Berkel 2014 | Not study related | Ana de Sena Cortabitarte, A., Degenhardt, F., Strohmaier, J., Lang, M., Weiss, B., Roeth, R., Giegling, I., Heilmann-Heimbach, S., Hofmann, A., Rujescu, D., Fischer, C., Rietschel, M., Nöthen, M. M., Rappold, G.A., & Berkel, S. Investigation of SHANK3 in schizophrenia. Am J Med Genet B *Neuropsychiatr. Genet*. **174**, 390–398 (2017). https://doi.org/10.1002/ajmg.b.32528. |
| 22 | Fuxe 2014 | Conference abstract | Fuxe, K., Borroto-Escuela, D. O., Guidolin, D., Tarakanov, A. O. & Agnati, L. F. The balance and integration of different forms of volume and wiring transmission in the CNS. Relevance for schizophrenia. *Neurology, Psychiatry and Brain Research*, **20,** 11–12 (2014). https://doi.org/10.1016/J.NPBR.2014.01.150 |
| 23 | Marion Schneider 2014 | Conference abstract | Marion Schneider, E., Karl Bechter., Hervé Perron. Cytoplasmic and microparticle-associated endogenous retrovirus Type W (HERV-W) in immature dendritic cells of patients with psychiatric disorders. *Neurology, Psychiatry and Brain Research* **20**,21(2014). https://doi.org/10.1016/j.npbr.2014.01.166. |
| 24 | De Smaele 2010 | Review | De Smaele, E,, Ferretti, E., Gulino, A. MicroRNAs as biomarkers for CNS cancer and other disorders. *Brain Res*, **1338,**100-11(2010). https://doi:10.1016/j.brainres.2010.03.103. |
| 25 | Wang 2022 | Review | Wang, Y., Amdanee, N., Zhang, X. Exosomes in schizophrenia: Pathophysiological mechanisms, biomarkers, and therapeutic targets. *Eur Psychiatry*. **65,** e61(2022). https://doi: 10.1192/j.eurpsy.2022.2319. |
| 26 | Szepesi 2018 | Review | Szepesi, Z., Manouchehrian, O., Bachiller, S., Deierborg, T. Bidirectional Microglia-Neuron Communication in Health and Disease. *Front Cell Neurosci*.**12,**323(2018). https://doi: 10.3389/fncel.2018.00323. |
| 27 | Nascimento 2016 | Review | Nascimento, J,M., Garcia, S., Saia-Cereda, V.M., Santana, A,G., Brandao-Teles, C., Zuccoli, G.S., Junqueira, D.G., Reis-de-Oliveira, G., Baldasso, P.A., Cassoli, J,S., Martins-de-Souza, D. Proteomics and molecular tools for unveiling missing links in the biochemical understanding of schizophrenia. *Proteomics Clin Appl* .**10,** 1148-1158(2016). https://doi: 10.1002/prca.201600021. |
| 28 | Smith 2017 | Review | Smith, M. DNA Sequence Analysis in Clinical Medicine, Proceeding Cautiously. *Front Mol Biosci*.**4,**24(2017). https://doi: 10.3389/fmolb.2017.00024. |
| 29 | Ilgın 2018 | Review | Ilgın, C., Topuzoğlu, A. Extracellular Vesicles in Psychiatry Research in the Context of RDoC Criteria. *Psychiatry Investig*.**15**,1011-1018(2018). https://doi: 10.30773/pi.2018.09.17. |
| 30 | Mobarrez 2013 | Case report | Mobarrez, F., Nybom, R., Johansson, V., Hultman, C.M., Wallén, H., Landén, M., Wetterberg, L. Microparticles and microscopic structures in three fractions of fresh cerebrospinal fluid in schizophrenia: case report of twins. *Schizophr Res*.**143,**192-7(2013). https://doi: 10.1016/j.schres.2012.10.030. |
| 31 | Hadas Tsivion-Visbord 2020 | Invitro-animal | Tsivion-Visbord, H., Perets, N., Sofer, T., Bikovski, L., Goldshmit, Y., Ruban, A., Offen, D. Mesenchymal stem cells derived extracellular vesicles improve behavioral and biochemical deficits in a phencyclidine model of schizophrenia. *Transl Psychiatry* **10,** 305(2020). https://doi: 10.1038/s41398-020-00988-y. Erratum in: *Transl Psychiatry* **10**,327(2020). https://doi: 10.1038/s41398-020-01016-9. Erratum in: *Transl Psychiatry* **10**,341(2020). https://doi: 10.1038/s41398-020-01030-x. |
| 32 | Zhang 2022 | Review | Zhang, Y., Xu, C. Effects of exosomes on adult hippocampal neurogenesis and neuropsychiatric disorders. *Mol Biol Rep*. **49,** 6763-6777(2022). https://doi: 10.1007/s11033-022-07313-4. |
| 33 | Zhang 2023 | Review | Zhang, T., Fang, Y., Wang, L., Gu, L., Tang, J. Exosome and exosomal contents in schizophrenia. *J Psychiatr Res*.**163**,365-371(2023). https://doi: 10.1016/j.jpsychires.2023.05.072. |
| 34 | Abdelaal 2021 | Book chapter/not focussed on schizophrenia | Abdelaal, N.E., & Abdelhai, M.F. Differential expression of exosomal microRNAs in neurodegenerative diseases. In: Alzahrani FA, Saadeldin IM, editors. *Role of exosomes in biological communication systems* (Springer, Singapore, 2021). https://doi.org/10.1007/978-981-15-6599-1_9 |
| 35 | Raghavan 2017 | Hypothesis | Raghavan, V., Bhomia, M., Torres, I., Jain, S., Wang, K.K. Hypothesis: Exosomal microRNAs as potential biomarkers for schizophrenia. *Med Hypotheses*,**103,**21-25(2017). https://doi: 10.1016/j.mehy.2017.04.003. |
| 36 | Duarte-Silva 2022 | Review | Duarte-Silva, E., Oriá, A.C., Mendonça, I.P., de Melo, M.G., Paiva, I.H.R., Maes, M, Joca, S.R.L., Peixoto, C.A. Tiny in size, big in impact: Extracellular vesicles as modulators of mood, anxiety and neurodevelopmental disorders. *Neurosci Biobehav* Rev. **135**,104582(2022). https://doi: 10.1016/j.neubiorev.2022.104582. |
| 37 | Peferoen 2014 | Review | Peferoen, L., Kipp, M., van der Valk, P., van Noort, J.M., Amor, S. Oligodendrocyte-microglia cross-talk in the central nervous system. *Immunology* **141,** 302-13(2014). https://doi: 10.1111/imm.12163. |
| 38 | Miller 2019 | Commentary | Miller, B. Brain Glucose, Insulin Resistance, and Memory in Schizophrenia. *Psychiatric Times* **36**, 13(2019). |
| 39 | Mauer 2021 | Conference abstract | Mauer, J., Xavier, G., Ota, V.K., Costa, G.O., Asprino, P.F., Novaes, A. et al. W70. Small rna expression profile from exosomes of first episode psychosis patients. *European Neuropsychopharmacology* **51**, 181-182(2021). https://doi.org/10.1016/j.euroneuro.2021.08.155. |
| 40 | Wijtenburg 2019 | Not relevant to research question | Wijtenburg, S.A., Kapogiannis, D., Korenic, S.A., Mullins, R.J., Tran, J., Gaston, F.E., Chen, S., Mustapic, M., Hong, L.E., Rowland, L.M. Brain insulin resistance and altered brain glucose are related to memory impairments in schizophrenia. *Schizophr Res*. **208**, 324-330(2019). https://doi: 10.1016/j.schres.2019.01.031. |
| 41 | Amoah 2019 | Conference abstract | Amoah, S.K., Rodriguez, B.A., Yellowhair, T.R., Logothetis, C.N., Floruta, C., Alural, B. et al. T123. A Psychosis-Altered Glial-Produced miRNA Downregulates Neuronal Gene Expression via Exosomes. Biological Psychiatry **85**, S176-S177(2019). https://doi:10.1016/j.biopsych.2019.03.446 |
| 42 | Liang 2024 | Animal study | Liang,J., Chen, L., Li, Y., Chen, Y., Yuan, L., Qiu, Y., Ma, S., Fan, F., Cheng, Y. Unraveling the Prefrontal Cortex-Basolateral Amygdala Pathway's Role on Schizophrenia's Cognitive Impairments: A Multimodal Study in Patients and Mouse Models. *Schizophr Bull*. **50,** 913-923(2024). https://doi: 10.1093/schbul/sbae063. |
| 43 | Lee 2019 | Conference abstract | Lee, E., Rissman, R., and Jeste, D. T227.Plasma exosomal amyloid and tau biomarkers in schizophrenia and non-psychiatric comparison subjects: Relationships with cognitive, mental, and physical functioning. *Neuropsychopharmacology* **44,** 350-351(2019). https://doi.org/10.1038/s41386-019-0546-x. |
| 44 | Dai 2023 | Review | Dai,J., Zhang, M.Z., He, Q.Q., Chen, R. The emerging role of exosomes in Schizophrenia. *Psychiatry Res*. **327**,115394(2023). https://doi: 10.1016/j.psychres.2023.115394. |
| 45 | Oraki 2023 | Review | Oraki Kohshour, M., Papiol, S., Delalle, I., Rossner, M.J., Schulze, T.G. Extracellular vesicle approach to major psychiatric disorders. *Eur Arch Psychiatry Clin Neurosci*. **273,** 1279-1293(2023). https://doi: 10.1007/s00406-022-01497-3. |
| 46 | Amoah 2023 | Poster | Amoah, Stephen. et al.2023 P1029 / #1670. Impact of antipsychotics on exosome secreted microRNA, mir-223 on glutamate receptor expression in neurons and astrocytes. [*IBRO Neuroscience Reports*](https://www.sciencedirect.com/journal/ibro-neuroscience-reports) [**15**](15), S526(2023). https://doi.org/10.1016/j.ibneur.2023.08.1035. |
| 47 | O'Brien 2016 | Doctoral thesis | O Brien. Bipolar disorder related functional variants in the calcium channel gene family. Doctoral thesis , UCL (University College, London) |
| 48 | Hewitt 2024 | Comment | Hewitt, K., Huang, X.F. Comment on 'Impact of clozapine on the expression of miR-675-3p in plasma exosomes derived from patients with schizophrenia'. *World J Biol* *Psychiatry* **25,** 281-282(2024). https://doi: 10.1080/15622975.2024.2349065. |
| 49 | Morgunova 2024 | Editorial | Morgunova, A., Teixeira, M., Flores, C. Perspective on adolescent psychiatric illness and emerging role of microRNAs as biomarkers of risk. *J Psychiatry Neurosci*. **49,** E282-E288(2024). https://doi: 10.1503/jpn.240072. |
| 50 | Wang 2024 | Animal study | Wang, Z., Wu, T., Hu, H., Alabed, A.A.A., Cui, G., Sun, L., Sun, Z., Wang, Y., Li, P. Plasma exosomes carrying mmu-miR-146a-5p and Notch signalling pathway-mediated synaptic activity in schizophrenia. *J Psychiatry Neurosci*. **49,** E265-E281(2024). https://doi: 10.1503/jpn.230118. |
| 51 | Zhong 2024 | Animal study | Zhong, X.L., Huang, Y., Du, Y., He, L.Z., Chen, Y.W., Cheng, Y., Liu, H. Unlocking the Therapeutic Potential of Exosomes Derived From Nasal Olfactory Mucosal Mesenchymal Stem Cells: Restoring Synaptic Plasticity, Neurogenesis, and Neuroinflammation in Schizophrenia. *Schizophr Bull*. **50,** 600-614(2024). https://doi: 10.1093/schbul/sbad172. |
| 52 | Smirnova 2024 | Not focussed on schizophrenia | Smirnova, L., Modafferi, S., Schlett, C., Osborne, L.M., Payne, J.L., Sabunciyan, S. Blood extracellular vesicles carrying brain-specific mRNAs are potential biomarkers for detecting gene expression changes in the female brain. *Mol Psychiatry*. **29,** 962-973(2024). https://doi: 10.1038/s41380-023-02384-6. |
| 53 | Wang 2023 | Review | Wang, X., Yang, H., Liu, C., Liu, K. A new diagnostic tool for brain disorders: extracellular vesicles derived from neuron, astrocyte, and oligodendrocyte. *Front Mol* *Neurosci.* **16**,1194210(2023). https://doi: 10.3389/fnmol.2023.1194210. |

**Supplementary Table S6. Conflict of Interest and Funding Source of studies**

| **Study ID** | **Conflict of Interest** | **Funding** |
| --- | --- | --- |
| Benigan 2013 | None Declared | This work was supported by the National Institute of Mental Health (R21 MH086079; ID and CRV) and National Institute of Aging (T32 AG00015-21; PFK). The funders had no role in study design, data collection and analysis, decision to publish, or preparation of the manuscript. |
| Amoah 2020 | None declared | This work was supported by a mentored PI grant as part of a P20 grant from the NIGMS (1P20GM121176-01—N.M. and S.K.A.). This work was supported in part by Dedicated Health Research Funds from the University of  New Mexico School of Medicine (N.M) |
| Du 2019 | None declared | National Natural Science Foundation of China (81703492); Beijing Natural Science Foundation (7182092); Minzu University Research Fund (2018CXTD03); Minzu University of China 111 project |
| Funahashi 2023 | None declared | This work was partially supported by a Health and Labour Science Research Grant from the Japanese Ministry of Health, Labour and Welfare and a Grant-in-Aid for Scientific Research from the Japanese Ministry of Education, Culture, Sports, Science and Technology (JSPS KAKENHI Grant No. 22K07597 and 22K07562). |
| Tomita 2023 | None declared | K.S., S.Y., S.A., M.M., M.I., K.K., A.N., and M.A. received grant support from the Japan Society for the Promotion of Science. K.K. received grant support from the MSD, Astellas, Sumitomo Dainippon Pharma, Eisai, Lily, Takeda, and Mitsubishi Tanabe Pharma. K.K. reports personal fees from Daiichi Sankyo, Otsuka, Meiji Seika Pharma, MSD, Yoshitomi, Astellas, Mochida, Sumitomo Dainippon, Eisai, and Fuji-Film RI Pharma outside the submitted work. All the other authors have no conflicts of interest to declare. |
| Tsoporis 2022 | None Declared | This work was supported by the Canadian Institutes of Health.Research (Grant # MOP-130331 to C.C.D.S. and MOP-111066 to T.G.P). |
| Khadimallah 2022 | A European Patent application has been filed (EP19218841.5) in relation to the development of diagnostic test derived from this work. KQD has received grant support not related to this work from Boehringer-Ingelheim. The other authors declared no other conflict of interest | National Center of Competence in Research (NCCR) “SYNAPSY-The Synaptic Bases of Mental Diseases” from the Swiss National Science Foundation (n°51AU40_125759 to KQD&PC), and Alamaya Foundation. Open Access funding provided by Université de Lausanne. |
| Tan 2021 | None | This study was supported by grants from the Natural Science Foundation of Guangdong (2018A030313427, 2017A030313490) and The Science and Technology Planning Project of Guangzhou City (202002030393). They were not involved in the design of the study and did not participate in the data collection, data analysis and manuscript writing. Mr. Yuanqi Feng works for Epibiotek Co. Ltd. for his technical consultant |
| Chuang Guo 2022 | None | The study was funded by the National Natural Science Foundations of China (No. 81660234, 82060256), the Natural Science Foundation of Inner Mongolia (No. 2020MS08028) and the Scientific Research Project of Inner Mongolia University for Nationalities (NMDYB20038) |
| Ranganathan 2022 | None | This work was supported by unrestricted research funds (SNRGY), Department of Veteran’s Affairs (Mohini Ranganathan), Brain and Behavior Research Foundation (Suhas Ganesh) and St Joseph’s Foundation. (T. Mohanakumar) |
| Lee 2021 | None declared | This study was supported, in part, by the National Institute of Mental Health [NIMH K23MH119375-01 (PI: EEL), T32 Geriatric Mental Health Program MH019934 and R01MH094151-01 (PI: DVJ)], NARSAD Young Investigator grant from the Brain and Behavior Research Foundation (PI: EEL), the National Institute on Aging P30-AG062429, R01AG051848, and R01AG058252 (PI: RAR), and National Institute of Neurological Disorders and Stroke [Diversity Supplement (PI: CW-G)], the VA San Diego Healthcare System, and the Stein Institute for Research on Aging (Director: DVJ) at the University of California San Diego. |
| Goetzl 2022 | Dr. Goetzl has filed an application with the US Office of Patents and Trademarks for the exosome methodology used in this research; no other authors have any conflicts of interest | HHS \| NIH \| National Institute of Mental Health (NIMH), Grant/Award Number: 01MH103831; HHS \| NIH \| National Institute on Aging (NIA), Grant/Award Number: Intramural |
| Goetzl 2021 | Dr. Goetzl has filed an application with the US Office of Patents and Trademarks for the exosome methodology used in this research; no other authors have any conflicts of interest | The study was supported in part by grant MH103831 from the National Institutes of Health (Srihari) |
| Goetzl 2020 | Dr. Goetzl has filed an application with the US Office of Patents and Trademarks for the exosome methodology used in this research; no other authors have any conflicts of interest | The study were supported in part by grant MH103831 from the National Institutes of Health (Srihari) |
| Kapogiannis 2019 | None declared | The research was supported in part by the Intramural Research Program of the National Institute on Aging, National Institutes of Health (Dimitrios Kapogiannis, Joyce Tran, Maja Mustapic). and by the German Federal Ministry of Education and Research (Marcella Rietschel 01EW1810) |
| Tunset 2020 | Hanne Haslene-Hox and Einar Sulheim are employed as Research Scientists at SINTEF, a non-profit research organization. | The work was supported by St. Olavs University Hospital (ref16/9564-97/NISLIN and ref17/10533-124/NISLIN) and by the Research Foundation—Flanders through an FWO SB PhD Grant (1S90918N). |
| Tunset 2023 | None declared | This work was supported by St. Olavs University Hospital (ref16/9564-97/ NISLIN, ref17/10533-124/NISLIN and 2020/13043-16) and by the Research Foundation—Flanders through an FWO SB PhD Grant (1S90918N). |
| Du 2021 | None declared | This study was supported by the National Natural Science Foundation of China (82071676 and 81703492), the Beijing Natural Science Foundation (7182092), the High-Level Hospital Development Program for Foshan “Climbing” Project, the Minzu University Research Fund (2018CXTD03), and the MUC 111 Project |
| Xue 2024 | None declared | The work was supported by National Key R&D Program of China  (2017YFC0909200 to D.H.C.); Shanghai Sailing Program  (17YF141630 to T.X.); National Natural Science Foundation of  China (82271539, 81801324 to T.X., 82271544 to D.H.C. and  82373937 to J.Y.); Shanghai Natural Science Foundation  (21ZR1455300 to T.X.); Shanghai Municipal Health Commission  Clinical Specialization in Health Industry (20214Y0057 to T.X.). |
| Barnett 2023 | M.M.B. and M.J.C. are inventors on Australian Provisional Patent  Application 2022903418, filed 14 November 2022, pertaining to the enrichment of neuronal  origin EVs from circulating tissues, an approach used in this study. W.R.R. and M.J.C. are  shareholders and director, respectively, of a company (PolygenRx Pty Ltd). The other authors  declare that they have no competing interests. There are no other patents to disclose | This work as supported by the National Health and Medical Research Council grant 1188493 and 1121474 (M.J.C.) and Australian Government Research Training Program stipend (M.M.B.). The ASRB was supported by National Health and  Medical Research Council, The Pratt Foundation, Ramsay Health Care, Viertel Charitable Foundation, and Schizophrenia Research Institute |
| Du 2023 | None declared | This work was funded by a grant from the National Natural Science Foundation  of China (grant numbers 81971601 to Yong Xu, 82271546 to Sha Liu),  Shanxi Scholarship Council of China(2022–190 to Sha Liu), Fundamental  Research Program of Shanxi Province (20210302123450 to Sha Liu,  202203021212038 to Xinzhe Du) and Scientific Research Project of Health  Commission of Shanxi Province (2021045 to Xinzhe Du). |
| Lorca 2024 | None declared | Support for this work was provided by the National Institute of Health/Instituto de Salud Carlos III-ISCIII, Spain (PI22/00443 to X.G.-P.) (grant co-funded by the European Union); the Ministry of Science and Innovation-MCIN, Spain and the National Research Council/Agencia Estatal de Investigación-AEI, Spain (PID2020-114885RB-C21 to A.S.) funded byMCIN/AEI/10.13039/501100011033.  This research was also co-financed by the Spanish Ministry of Science and Innovation with funds from the European Union Next Generation EU; from the Recovery, Transformation and Resilience Plan (PRTR-C17.I1); and from the Autonomous Community of Catalonia within the framework of the Biotechnology Plan Applied to Health ((EVBRAINTARGET-Y7340-ACPPCCOL007 to X.G.-P., A.S., M.Mur, and A.R.-M.) coordinated by the Institute for Bioengineering of Catalonia (IBEC)); the Diputació de Lleida, Spain (PIRS22/03 to X.G.-P. & I.B. and PIRS23/02 to A.S.); the Catalan Research Council-AGAUR (AGAUR 21SGR010065 to E.V.; 2023 LLAV 00056 to X.G.-P.; and 2022 DI 100 to X.G.-P.); and the Basque Government (IT211/19 and IT1512/22 to J.J.M and L.F.C.). X.G.- P. acknowledges a Miguel Servet program tenure track contract (CP21/00096) from the ISCIII, awarded on the 2021 call under the Health Strategy Action, co-funded by the European Union (FSE+). A.S. acknowledges a Ramón y Cajal program tenure track contract (RYC2021-030946-I) funded by MCIN/AEI/10.13039/501100011033 and by the “European Union NextGenerationEU/PRTR”; A.R.-M. acknowledges a Ramón y Cajal program tenure track contract (RYC-2016-19282) funded by MCIN/AEI/10.13039/501100011033. M.F.-R.’s postdoctoral contract is funded by PRTR-C17.I1 and EVBRAINTARGET-Y7340-ACPPCCOL007. C.L.’s PhD is funded by the European Social Fund for the recruitment of predoctoral researchers (PEJD-2019-PRE/BIO-16475); M.M.’s PhD is funded by the MCIN-AEI (PR2021-097934); and J.A.S.M.’s PhD is funded by AGAUR (2023 FI-1 00054), and J.A.S.M.’s contributions were also supported by Diputació de Lleida ‘Ajuts al Talent en Investigació Biomèdica”. IRBLLEIDA, J.A.S.M., X.G.-P., and A.S. are co-funded by the CERCA Program/ Generalitat de Catalunya. J.J.M., A.R.-M., and X.G.-P. are members of the ExoPsyCog Consortium,  funded by IKUR-Neurobiosciences—Basque Government. |
| Xu 2024 | None declared | This work was supported by the National Natural Science  Foundation of China (grant no. 82071676) and the Medical Science  and Technology Research Fund Project of Guangdong Province (grant  no. A2024206) |
| Zhang 2024 | None declared | This work was supported by the National Key Research and Development Program of  China (Grant No. 2021YFE0191400 (X.C.)), National Natural Science Foundation of  China (Grant No. 82101576 (Z.L.)), Science and Technology Innovation Program of  Hunan Province (Grant No. 2022RC1040 (Z.L.)), Natural Science Foundation of Hunan Province of China (Grant No.2022JJ40695 (Z.L.)), the Scientific Research Launch Project for new employees of the Second Xiangya Hospital of Central South  University (Z.L. and Y.H.), and the Fundamental Research Funds for the Central  Universities of Central South University (Grant No. 2024ZZTS0932 (S.Z.)) |
